# Basal body docking failure triggers centriole clustering and elimination in mammalian spermatogenesis

**DOI:** 10.1038/s44319-026-00872-8

**Published:** 2026-07-18

**Authors:** Jun Jie Chen, Xiangyu Gong, Michael Mak, Feng-Qian Li, Ken-Ichi Takemaru

**Affiliations:** 1 Graduate Program in Molecular and Cellular Biology, Stony Brook University, Stony Brook, NY USA; 2 Department of Pharmacological Sciences, Stony Brook University, Stony Brook, NY USA

**Keywords:** Cell Adhesion, Polarity & Cytoskeleton, Development, Organelles

## Abstract

Cilia are microtubule-based organelles essential for signaling and motility, and their dysfunction causes ciliopathies often associated with infertility. In male germ cells, two types of cilia are present: zygotene primary cilia and sperm flagella. To define the role of cilia in spermatogenesis, we conditionally ablate the distal appendage protein CEP164, required for basal body docking and ciliogenesis, in male germ cells. CEP164 localizes to the mother centriole/basal body throughout spermatogenesis, and its loss leads to male infertility accompanied by the absence of both zygotene cilia and sperm flagella. Despite defective ciliogenesis, meiotic chromosome pairing and DNA double-strand break repair proceed normally. However, round spermatids exhibit basal body docking and flagellogenesis defects, and frequently form supernumerary centriole clusters, which appear to be subsequently eliminated via residual bodies. Live-cell imaging reveals that centrioles are highly mobile, and centriole pairs from neighboring cells are often associated, possibly through intercellular bridges, forming aggregates. These results establish that basal body docking is crucial for retaining centrioles within spermatids, and its disruption leads to centriole clustering and loss.

## Synopsis

CEP164-mediated basal body docking is required for the formation of zygotene cilia and sperm flagella during mammalian spermatogenesis. In contrast to previous findings in zebrafish, loss of zygotene cilia in mice does not overtly impair meiotic DNA repair or homologous chromosome synapsis, whereas defective basal body docking in spermatids induces centriole clustering and elimination, highlighting a critical role in centriole retention.

- CEP164 is essential for flagellogenesis and male fertility in mice.
- Zygotene cilia are dispensable for meiotic progression during mammalian spermatogenesis.
- Impaired basal body docking renders undocked centrioles highly dynamic and mobile, forming abnormal clusters in spermatids potentially via intercellular bridges.
- Supernumerary centrioles appear to be eliminated from elongated spermatids through residual bodies.

## Abstract (web summary)

CEP164-mediated basal body docking is required for the formation of zygotene cilia and sperm flagella during mammalian spermatogenesis. In contrast to previous findings in zebrafish, loss of zygotene cilia in mice does not overtly impair meiotic DNA repair or homologous chromosome synapsis, whereas defective basal body docking in spermatids induces centriole clustering and elimination, highlighting a critical role in centriole retention.

## Graphical abstract

**Figure:**
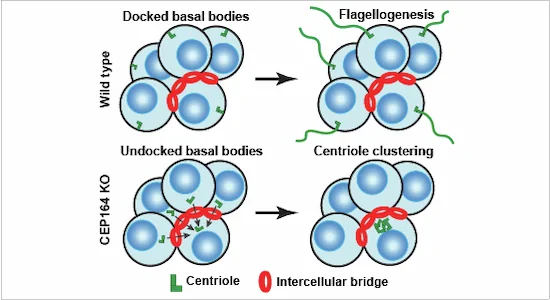

**Figure:**
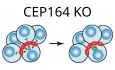

## Introduction

Cilia are highly conserved, microtubule-based organelles that extend from the surface of many different cell types (Hilgendorf et al, 2024; Hyland and Brody, 2021; Mill et al, 2023; Moran et al, 2024; Wallmeier et al, 2020). Based on their axonemal architecture, cilia are broadly classified into two types: non-motile primary cilia, with a 9 + 0 microtubule arrangement, and motile multicilia, with a 9 + 2 arrangement. Primary cilia are ubiquitous and function as sensory organelles that respond to chemical and mechanical cues in the environment. In contrast, multicilia are restricted to specialized epithelia, such as those of the airways, reproductive tracts, and the brain ventricles, where they generate directional fluid flow through coordinated beating. Sperm flagella represent a unique variant: singular motile organelles with a 9 + 2 axoneme and elaborate accessory structures that are indispensable for motility. Dysregulation of cilia causes a spectrum of multisystemic disorders known as ciliopathies, which frequently include male infertility. To date, mutations in at least 247 genes have been associated with ciliopathies, affecting either or both ciliary types (Mill et al, 2023; Moran et al, 2024).

Ciliogenesis is initiated during the G0/G1 phase of the cell cycle, when the mother centriole transforms into a basal body that first engages with small vesicles or the plasma membrane prior to axoneme assembly (Hilgendorf et al, 2024; Mill et al, 2023; Sorokin, 1968). This direct basal body-membrane docking is mediated by distal appendages, pinwheel-like fibrous structures emanating from each microtubule triplet (Kanie et al, 2025; Ma et al, 2023). Distal appendages are composed of several core proteins, including centrosomal protein of 164 kDa (CEP164). CEP164 is critical for small vesicle recruitment and basal body docking during both primary and multiciliogenesis (Schmidt et al, 2012; Siller et al, 2017). CEP164 recruits downstream ciliary proteins, including Cby1 and ciBAR family members, to promote basal body docking and ciliogenesis (Burke et al, 2014; Hoque et al, 2021; Li et al, 2016; Siller et al, 2017). We previously demonstrated that multiciliated cell-specific *Cep164*-knockout (KO) mice exhibit a profound loss of multicilia in the trachea, oviduct, and ependyma, with ~20% succumbing to severe hydrocephalus (Siller et al, 2017). More recently, we reported that male mice are infertile due to dysfunctional multicilia in the efferent ducts connecting the testis and epididymis (Hoque et al, 2021). In striking contrast to its essential role in mammalian ciliogenesis, the *Drosophila* CEP164 ortholog is dispensable for basal body docking, ciliogenesis, and male fertility (Hou et al, 2023). However, the role of CEP164 in mammalian spermatogenesis has remained unknown. Beyond its ciliary functions, CEP164 has also been implicated in DNA damage responses (Chaki et al, 2012; Pan and Lee, 2009), although this role has been challenged (Daly et al, 2016).

Mammalian spermatogenesis is a complex and tightly regulated process that generates haploid gametes (spermatozoa) from diploid spermatogonial stem cells within the seminiferous tubules of the testis (Griswold, 2016; Han, 2024; Teves and Roldan, 2022). In mice, the process is organized into 12 stages and completed in ~34.5 days (Oakberg, 1956). Spermatogenesis can be broadly categorized into three major phases: (1) self-renewal of spermatogonial stem cells, (2) meiosis, in which spermatocytes give rise to spermatids, and (3) spermiogenesis, the differentiation of spermatids into mature spermatozoa, which proceeds through 16 defined steps, including flagellogenesis. A unique feature of both male and female gametogenesis is the formation of stable intercellular bridges that physically interconnect developing germ cells, creating a syncytial network (Gerhold et al, 2022; Greenbaum et al, 2011). The diameter of these cytoplasmic bridges can reach up to 3.0 μm, allowing the exchange of RNAs, proteins, and organelles, thereby ensuring their synchronized development and functional coordination within the cyst (Greenbaum et al, 2011).

Recent studies in zebrafish have reported the presence of primary cilia in both primary oocytes and spermatocytes (Mytlis et al, 2022; Xie et al, 2022). In oocytes, primary cilia are detected at leptotene and zygotene stages and are essential for proper formation of the meiotic chromosomal bouquet, an evolutionarily conserved configuration that facilitates synaptonemal complex assembly and homologous chromosome pairing (Mytlis et al, 2022; Mytlis et al, 2023). During bouquet formation, telomeres slide along perinuclear microtubules and cluster at a small region of the inner nuclear membrane, creating the “floral arrangement”, hence the name. Strikingly, loss of zygotene cilia disrupts synaptonemal complex formation, impairs oogenesis, and leads to infertility. However, this conclusion is in contrast to previous findings indicating that germ cell-specific removal of cilia exhibits no significant effects on oogenesis and fertility in zebrafish females, while causing infertility in males (Borovina and Ciruna, 2013; Liu et al, 2023; Xie et al, 2022). In zebrafish spermatocytes, primary cilia are observed from leptotene through diplotene stages (Xie et al, 2022). Germ cell-specific ablation of primary cilia results in defective DNA double-strand break repair (DSB), increased apoptosis, and infertility in males. In mice, primary cilia are detected at the zygotene stage in the adult testis (Lopez-Jimenez et al, 2022). Notably, only about 20% of zygotene spermatocytes extend primary cilia. Analysis of the timing of ciliogenesis relative to bouquet formation indicated that primary cilia are not directly involved in bouquet formation, suggesting that zygotene cilia are dispensable for homologous chromosome pairing in mammalian spermatocytes. Nevertheless, the role of primary cilia in mammalian germ cells remains to be elucidated.

To investigate the role of CEP164 and primary cilia in mammalian spermatogenesis, we generated male germ cell-specific *Cep164*-KO mice. We found that cilia were largely absent in zygotene spermatocytes and sperm, and males were infertile. Notably, meiotic chromosome pairing and DSB repair progressed normally in the absence of primary cilia. In contrast, loss of CEP164 caused impaired basal body docking and defective flagellogenesis in spermatids. Unexpectedly, undocked centrioles were highly mobile and associated with one another, likely through intercellular bridges, to form aggregates that appeared to be ultimately discarded into residual bodies in the lumen of seminiferous tubules. In sum, our findings demonstrate that basal body docking mediated by CEP164 is essential for centriole immobilization and retention, and flagellogenesis, whereas zygotene cilia are dispensable for meiotic chromosome pairing or DNA repair during mammalian spermatogenesis.

## Results

### Loss of CEP164 in male germ cells causes infertility

To investigate the biological role of cilia in spermatogenesis, we conditionally deleted *Cep164* in male germ cells by crossing *Cep164*^*fl/fl*^ mice (Hoque et al, 2021; Siller et al, 2017) with *Stra8*-iCre, which transiently expresses Cre recombinase from type A spermatogonia to preleptotene spermatocytes (Sadate-Ngatchou et al, 2008).

**Figure EV1:**
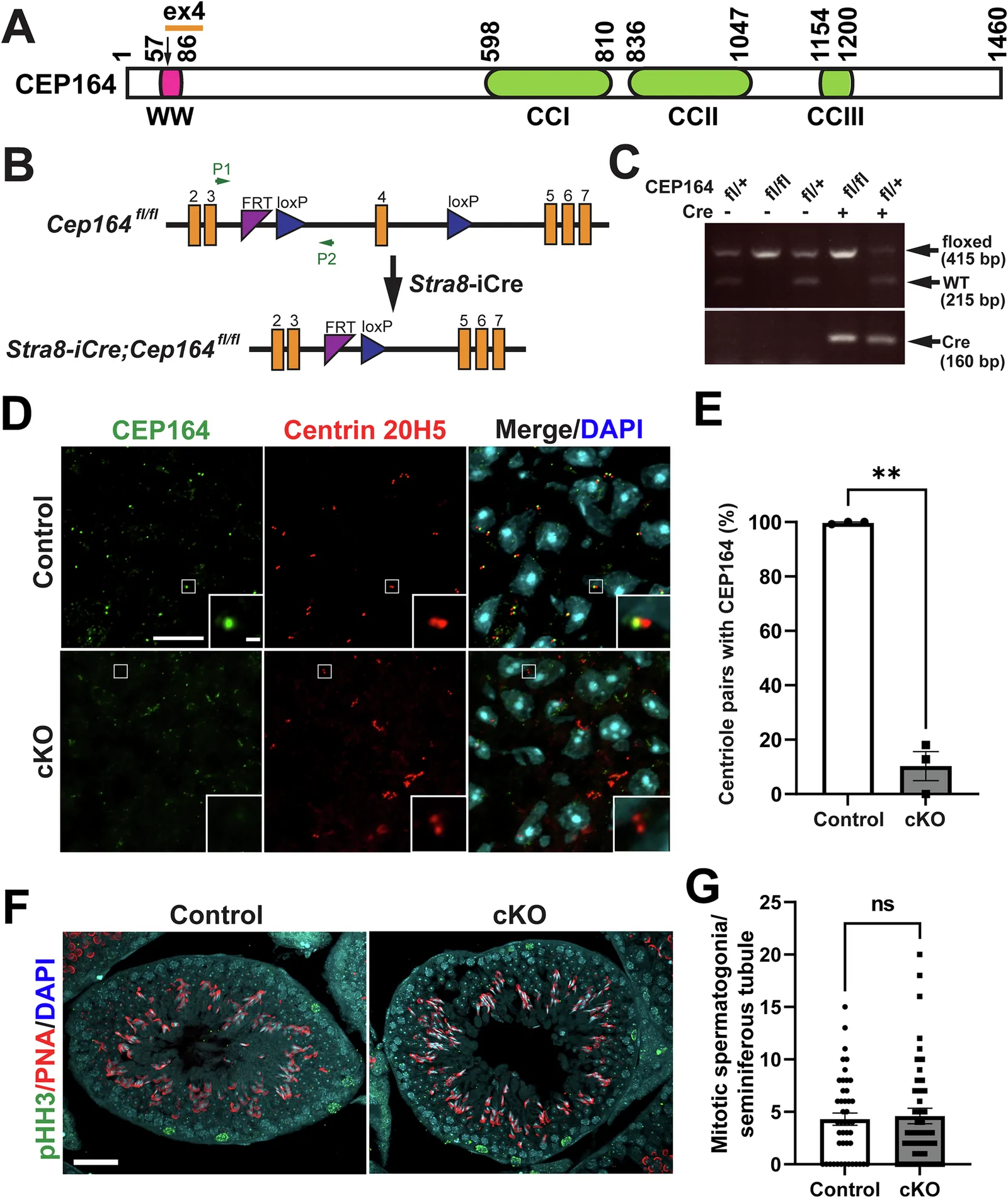
Generation of *Stra8-iCre;Cep164*^*fl/fl*^ mice. (**A**) Schematic of the CEP164 protein domain organization, showing a WW domain and three coiled-coil motifs (CCI-CCIII). The arrow marks the truncation site at amino acid 65 following Cre-mediated recombination. (**B**) Strategy for generating *Stra8-iCre;Cep164*^*fl/fl*^ mice. The locations of genotyping primers are indicated by green arrows (P1 and P2). (**C**) PCR-based mouse genotyping. (**D**) IF staining of P23 testis frozen sections using CEP164 and Centrin 20H5 antibodies. Nuclei were visualized by DAPI. Scale bars, 10 and 1 µm (insets). (**E**) Quantification of centriole pairs with CEP164 in round spermatids from control and cKO postnatal testes. *n* = 3 mice per genotype (one littermate pair each at P20, P22, and P23), with over 100 round spermatids counted per mouse. Round spermatids are abundantly present at P20-P23 during the first wave of spermatogenesis. Error bars represent ± SEM. Welch’s *t* test: **P = 0.0035. (**F**) IF staining of adult testis paraffin sections using phospho-histone H3 (Ser10) antibody, counterstained with PNA and DAPI. Scale bar, 10 µm. (**G**) Quantification of mitotic spermatogonia. n = 3 mice per genotype, with 40 tubules counted per mouse. Error bars represent ± SEM. Welch’s *t* test: not significant (ns), *P* = 0.7596. Source data are available online for this figure

CEP164 consists of 1,460 amino acids and contains a WW domain and three coiled-coil domains (Fig. EV1A). Cre-mediated recombination excises exon 4, resulting in a frameshift and premature truncation at amino acid 65 (Fig. EV1B). These mice are hereafter referred to as cKO. Genotypes were confirmed by PCR analysis of tail DNA (Fig. EV1C). Immunofluorescence (IF) staining showed that CEP164 was absent from the mother centriole in 89% of cKO spermatids, indicating efficient Cre-mediated recombination (Fig. EV1D,E).

Since *Stra8*-iCre is expressed in type A spermatogonia, and CEP164 depletion has been reported to affect the cell cycle in cultured mammalian cells (Slaats et al, 2014), we asked whether loss of CEP164 might also influence cell-cycle progression in spermatogonia and thereby affect downstream processes. To examine this, we performed IF staining for phospho-histone H3 (Ser10) (pHH3), a mitotic marker (Fig. EV1F). Quantification revealed that the mitotic index of spermatogonia was comparable between control and cKO testes (Fig. EV1G). Although we cannot fully exclude subtle effects, these data suggest that spermatogonial cell-cycle progression is not obviously affected by loss of CEP164.

**Figure 1:**
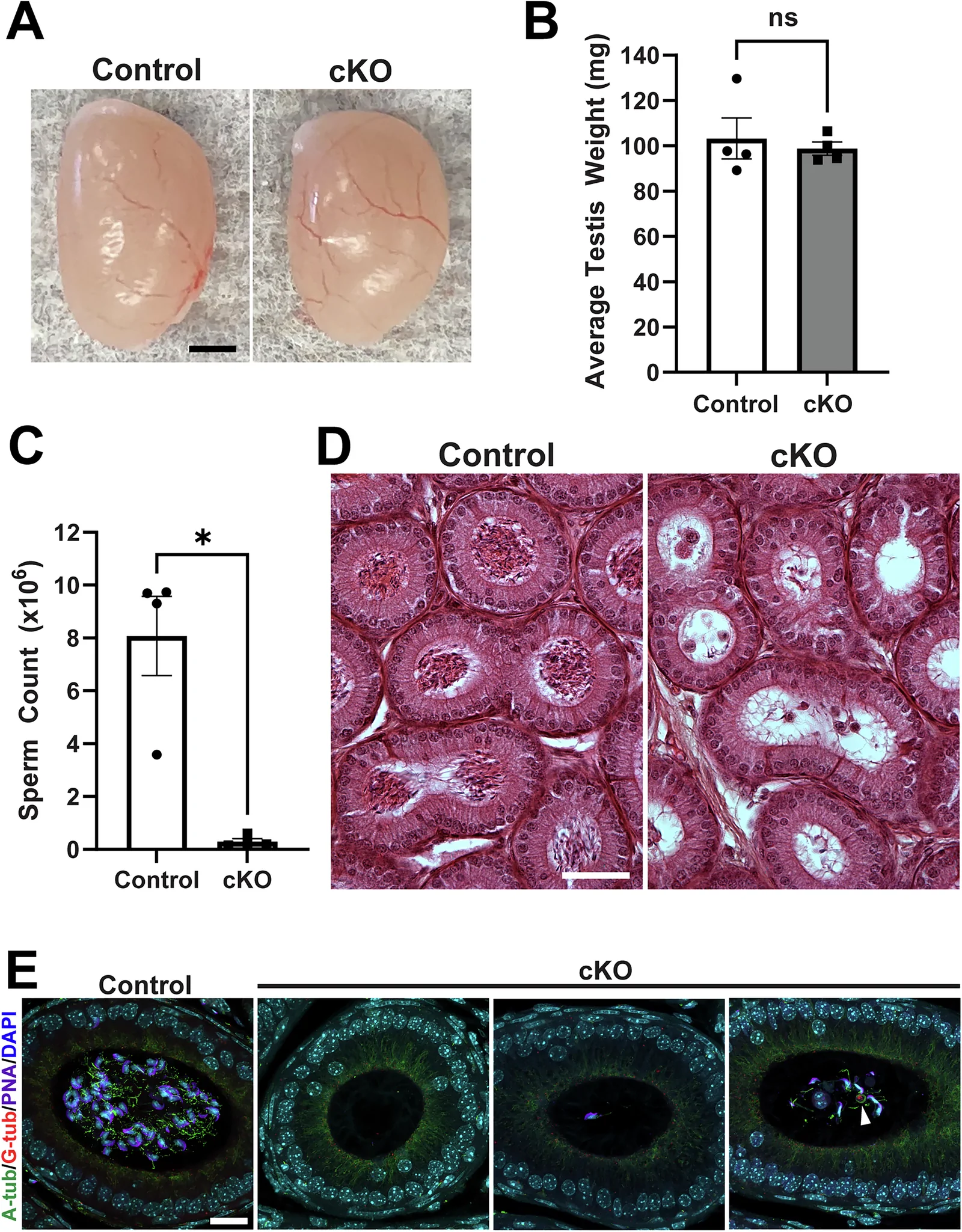
*Stra8-iCre;Cep164*^*fl/fl*^ males are infertile with reduced sperm counts. (**A**) Representative images of adult testes from *Cep164*^*fl/fl*^ (control) and *Stra8-iCre;Cep164*^*fl/fl*^ (cKO) mice. Scale bar, 2 mm. Quantification of testis weight (**B**) and sperm count (**C**). Adult mice (2–5-month-old) were euthanized, and testes were collected for weight measurements. Sperm were isolated from the cauda epididymis and counted using a hemocytometer. *n* = 4 per genotype. Error bars represent ± SEM. Welch’s *t* test: (**B**) not significant (ns), *P* = 0.6658; (**C**) *P = 0.0137. (**D**) Representative images showing H&E staining of adult caput epididymal sections. Scale bar, 50 µm. (**E**) IF staining of adult caput epididymal paraffin sections with antibodies against acetylated α-tubulin (A-tub; flagellar marker) and γ-tubulin (G-tub; centriole marker), together with lectin peanut agglutinin (PNA; acrosomal marker) and DAPI (nuclei) counterstaining. Arrowhead indicates a centriole cluster in an abnormal round cell (see below for details). Scale bar, 20 µm. Source data are available online for this figure.

To examine the biological importance of CEP164 during spermatogenesis, we evaluated male fertility by mating *Cep164*^*fl/fl*^ control and cKO males with *Cep164*^*fl/fl*^ control females. cKO males failed to produce any offspring (Table 1), indicating infertility. To shed light on the underlying cause, we examined testis morphology, weight, and sperm production. Testes from cKO mice appeared grossly normal (Fig. 1A), and testis weights were comparable to those of controls (Fig. 1B). However, cKO mice exhibited a dramatic reduction in the number of mature sperm harvested from the cauda epididymis (Fig. 1C). Consistent with this finding, hematoxylin and eosin (H&E) staining revealed an absence of mature sperm and the presence of abnormal round cells in the epididymal lumen of cKO males (Fig. 1D). Furthermore, IF staining of adult caput epididymal sections using antibodies against acetylated α-tubulin (henceforth A-tub; flagellar marker) and γ-tubulin (henceforth G-tub; centriole marker), together with lectin peanut agglutinin (PNA; acrosomal marker) and DAPI counterstaining confirmed that the cKO epididymis was largely devoid of mature sperm, with only rare sperm and occasional abnormal round cells detected (Fig. 1E). These results demonstrate that CEP164 is essential for male fertility in mice.

**Table 1:** Fertility test of Stra8-Cre;CEP164^fl/fl^ male mice.

Male genotype	Litter size
CEP164^fl/fl^	7.4 ± 0.3
Stra8-iCre;CEP164^fl/fl^	0.0

Five males for each genotype were each mated with CEP164^fl/fl^ females for about 2 months, and litter numbers were scored. Data are presented as mean ± SEM.

### Defective flagellogenesis and aberrant sperm head elongation in cKO testes

**Figure 2:**
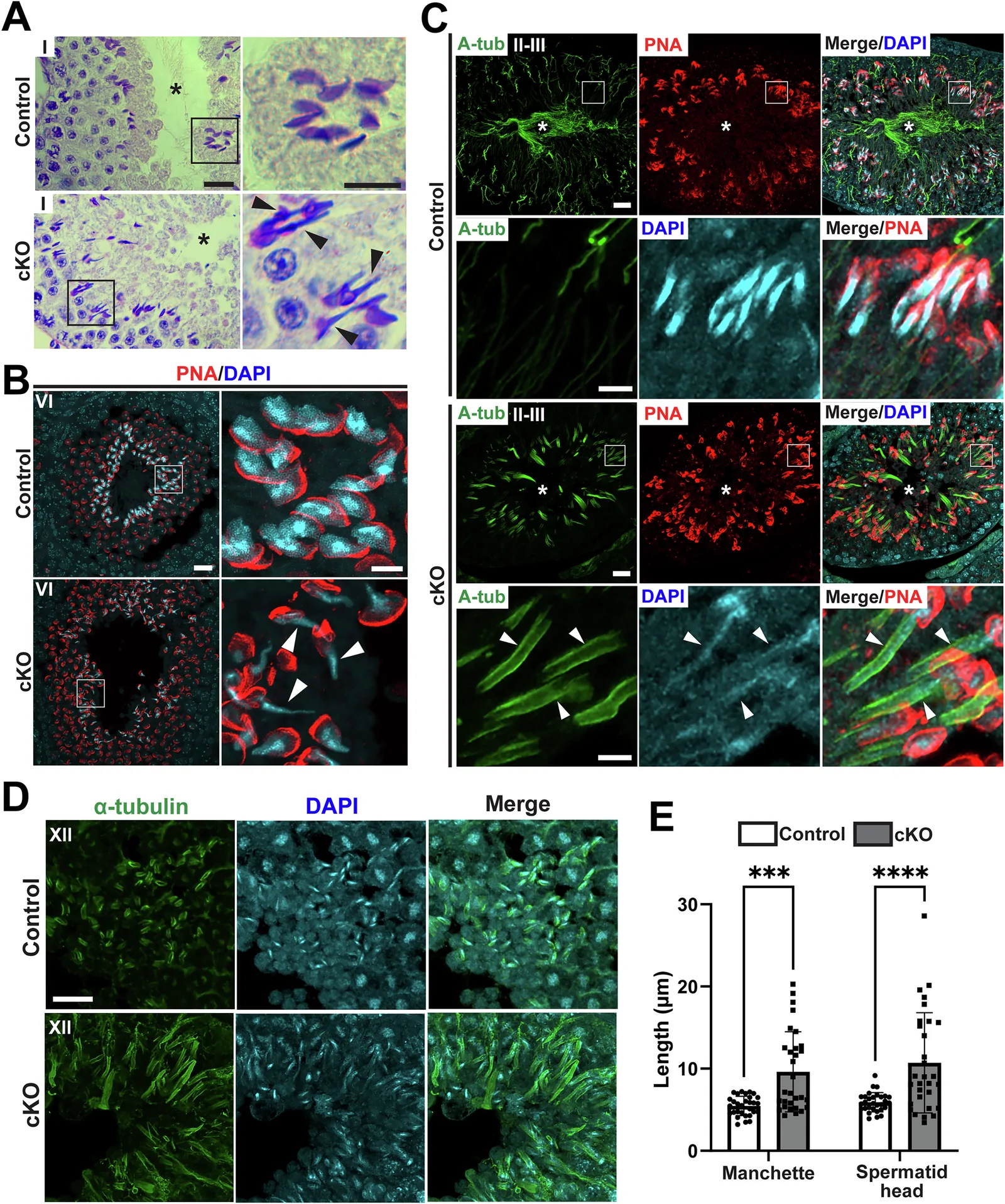
Histological and immunofluorescence analysis of testes. (**A**) Periodic acid-Schiff (PAS) staining of seminiferous tubules from adult control and cKO mice (spermatogenic stage I). Asterisks indicate the lumen where sperm flagella are typically observed. Arrowheads mark abnormally elongated sperm heads in cKO tubules. Scale bars, 20 and 10 µm (enlarged images). (**B**) Paraffin sections of adult testes stained with PNA and DAPI (stage VI). Arrowheads highlight abnormally elongated sperm heads in cKO tubules. Scale bars, 20 and 5 µm (enlarged images). (**C**) IF staining of adult testis paraffin sections with A-tub antibody (marker of flagella and stable microtubules), counterstained with PNA and DAPI (stage II–III). Asterisks denote the lumen. Arrowheads point to abnormally elongated sperm heads in cKO tubules with long manchettes in cKO images. Scale bars, 20 µm and 5 µm (enlarged images). (**D**) IF staining of adult testis paraffin sections for α-tubulin to visualize manchettes, with DAPI counterstain (stage XII). Stage XII tubules were identified based on the presence of spermatocytes undergoing meiotic division. Scale bar, 20 µm. (**E**) Quantification of manchette and spermatid head lengths at stage XII. *n* = 3 per genotype, with ten cells analyzed per mouse. Error bars represent ± SEM. Šidák multiple comparisons test: Manchette, ***P = 0.0002; Spermatid head, ****P = 0.000017. Source data are available online for this figure.

To gain insight into spermatogenesis defects in cKO mice, we performed periodic acid-Schiff (PAS) staining on adult testis sections. In *Cep164*^*fl/fl*^ control sections, elongated sperm flagella were readily observed in the lumen (Fig. 2A, asterisk). In marked contrast, cKO testes lacked detectable flagella. In addition, we observed abnormally elongated sperm heads in cKO sections (Fig. 2A, arrowheads). This was more clearly visualized by co-staining with PNA and DAPI (Fig. 2B, arrowheads). IF staining for A-tub (a marker of flagella and stable microtubules) further confirmed the absence of flagella in cKO seminiferous tubules (Fig. 2C, asterisk). Notably, the manchette, a transient microtubule structure required for sperm head shaping, was intensely labeled with A-tub antibody and appeared abnormally elongated in cKO spermatids (Fig. 2C, arrowheads), likely contributing to the excessively elongated head morphology. Similar elongated head structures have been reported in mouse models of ciliary defects (Lehti et al, 2013; San Agustin et al, 2015; Zhang et al, 2016). To quantify the degree of manchette and spermatid head elongation in the cKO testes, we performed IF imaging of adult testis sections stained for α-tubulin together with DAPI counterstaining to visualize the manchette and nuclei, respectively (Fig. 2D). At stage XII, when manchette elongation is nearly complete in controls, both the manchette and nuclei were significantly longer in the cKO samples (Fig. 2E). Taken together, these findings demonstrate that, in contrast to *Drosophila*, CEP164 is essential for mammalian spermatogenesis.

### Depletion of zygotene cilia does not significantly impair meiotic chromosome pairing or DNA repair during murine spermatogenesis

In zebrafish oogenesis, primary cilia in zygotene oocytes are required for meiotic bouquet formation and subsequent homologous chromosome pairing (Mytlis et al, 2022). Our cKO model provided us an opportunity to investigate the role of zygotene cilia in mouse spermatogenesis. To facilitate the visualization of centrioles, we utilized the ROSA-EGFP-Centrin1 mouse line, which ubiquitously expresses EGFP-Centrin1 in all cell types (Hirai et al, 2016).

**Figure 3:**
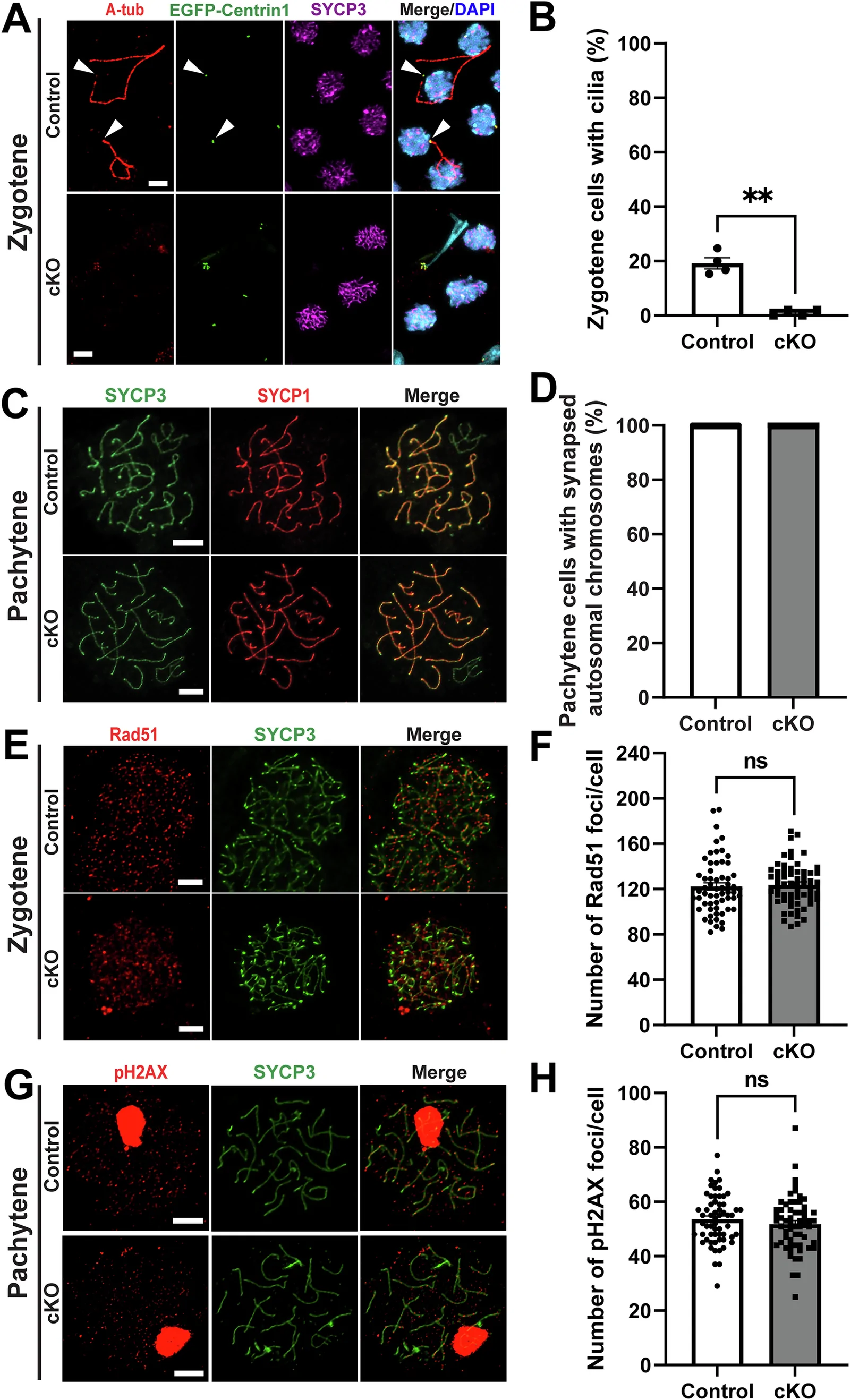
Loss of zygotene cilia does not overtly affect meiotic chromosome pairing or DNA repair. (**A**) IF staining of seminiferous tubule squashes from control or cKO mice expressing EGFP-Centrin1 (centriole marker) with antibodies against A-tub and SYCP3 (meiotic chromosomal axis marker). Arrowheads indicate basal bodies extending primary cilia in zygotene cells. Scale bar, 5 µm. (**B**) Quantification of zygotene cilia. *n* = 4 per genotype, with 150 zygotene cells scored per mouse. Error bars represent ± SEM. Welch’s *t* test: **P = 0.002. (**C**) IF staining of chromosome spread preparations for SYCP3 and SYCP1 (marker of synapsed regions of the synaptonemal complex). Scale bar, 5 µm. (**D**) Quantification of pachytene spermatocytes with fully synapsed autosomal chromosomes. Note that complete autosomal synapsis was observed in all 30 pachytene spermatocytes examined in both control and cKO testes, resulting in overlapping data points at 100% in the graph. *n* = 3 per genotype, with 10 cells analyzed per mouse. (**E**, **G**) IF staining of chromosome spread preparations of zygotene spermatocytes (**E**) for SYCP3 and Rad51 (DNA recombinase mediating homologous strand exchange) and (**G**) pachytene spermatocytes for SYCP3 and pH2AX (marker of DNA double-strand break sites). Scale bars, 5 µm. (**F**, **H**) Quantification of foci for each DNA repair marker. *n* = 4 per genotype, with 15 cells quantified per mouse. Error bars represent ± SEM. Welch’s *t* test: (**F**) not significant (ns), *P* = 0.7086; (**H**) ns, *P* = 0.3159. Note that variability in Rad51 and pH2AX foci size was observed in both control and cKO samples, and the images shown are representative. Source data are available online for this figure.

To quantify the proportion of zygotene spermatocytes bearing primary cilia, we prepared seminiferous tubule squashes from adult testes, followed by IF staining for A-tub and synaptonemal complex 3 (SYCP3; a meiotic chromosomal axis marker) (Fig. 3A). In control testes, primary cilia, typically long (8–25 μm) and emanating from centrioles (Fig. 3A, arrowheads), were detected in 19.2% (115/600) of zygotene spermatocytes (Fig. 3B), in agreement with previous observations (Lopez-Jimenez et al, 2022). In marked contrast, primary cilia were observed in only 1.0% (6/600) of cKO zygotene spermatocytes, indicating an approximately 95% reduction compared with controls.

Next, we evaluated whether loss of cilia influences meiotic chromosome pairing in cKO spermatocytes. Chromosome spread slides were prepared from dissociated seminiferous tubules and immunostained for SYCP1 and SYCP3 (Fig. 3C). SYCP3 decorates the lateral elements of both paired and unpaired chromosomal regions, whereas SYCP1 localizes to the central elements of paired chromosomes. We observed that all 19 autosomes were fully paired in all 30 pachytene spermatocytes that we examined in both control and cKO testes, indicating that homologous synapsis occurs normally without primary cilia (Fig. 3D).

During prophase I, homologous chromosome pairing is closely coordinated with DNA double-strand break (DSB) formation and repair. CEP164 has been implicated in DNA damage response pathways (Chaki et al, 2012; Sivasubramaniam et al, 2008), and in zebrafish, primary cilia play a critical role in meiotic homologous recombination repair in spermatocytes (Xie et al, 2022). To assess if cKO spermatocytes show defects in DSB repair processes, we performed IF staining for key DNA repair proteins: Rad51, a critical recombinase mediating homologous strand exchange, and phospho-histone H2A.X (γH2AX [Ser139]), which marks DSB sites. The number of foci for these markers was comparable between control and cKO spermatocytes (Fig. 3E–H), indicating that DSB repair was not significantly impaired. Thus, these results suggest that primary cilia, unlike in zebrafish, are not essential for meiotic chromosome pairing and DNA repair in the mouse testis.

### Basal body docking defects and centriole clustering in cKO spermatids

**Figure 4:**
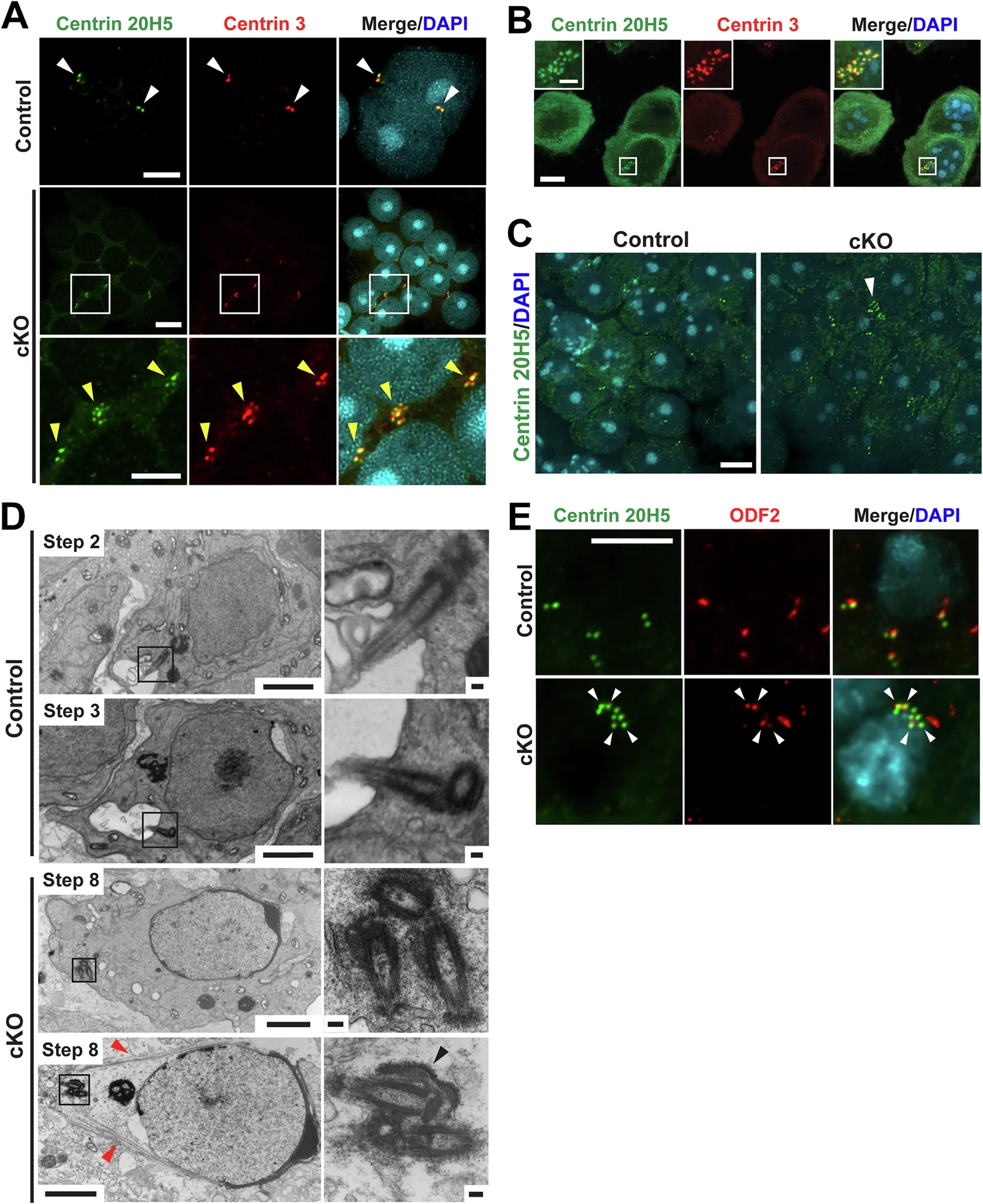
Abnormal clustering of supernumerary centrioles in cKO spermatids. (**A**, **B**) IF staining of chromosome spreads from cKO testes using Centrin 20H5 and Centrin 3 antibodies. (**A**) In control round spermatids, two centrioles are typically present (white arrowheads). In contrast, cKO round spermatids exhibit abnormal aggregates of multiple centrioles (yellow arrowheads). Enlarged views of boxed regions are shown below. Scale bars, 10 and 5 µm (enlarged images). (**B**) Representative example showing 23 centrioles within a single cKO round spermatid. Scale bars, 20 µm and 5 µm (insets). (**C**) IF staining of P22 testis paraffin sections using Centrin 20H5 antibody. The arrowhead indicates a centriole cluster. Scale bar, 5 µm. (**D**) TEM images of P26 testis sections. While control step 2–3 spermatids show docked centrioles with flagella, cKO step 8 spermatids display three undocked centrioles in a single cell. The red arrowhead indicates manchette microtubules. while the black arrowhead denotes basal plate-like structures typically present at the head-tail junction. Enlarged views of boxed regions are shown to the right. Scale bars, 2 µm and 100 nm (enlarged images). (**E**) IF staining of testis frozen sections using Centrin 20H5 and ODF2 (mother centriole marker) antibodies. Arrowheads point to mother centrioles. Scale bar, 10 µm. Source data are available online for this figure.

**Figure EV2:**
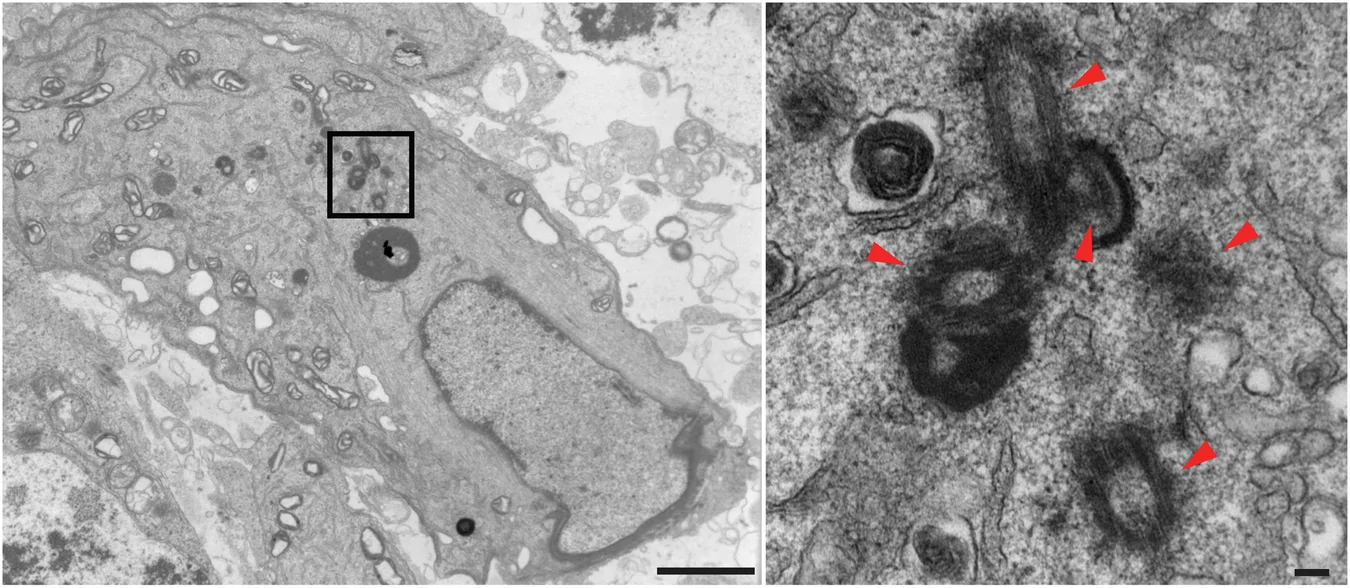
TEM image of undocked supernumerary centrioles in a cKO spermatid. TEM image of a P26 testis section showing a step 9 cKO spermatid containing five centrioles (red arrowheads). An enlarged view of the boxed region is shown on the right. Scale bars, 2 µm and 100 nm (enlarged images).

During the course of our study, we unexpectedly observed abnormal aggregation of multiple centrioles in cKO testes (Fig. 4A, yellow arrowheads). To ensure detection accuracy, centrioles were visualized using two independent centrin antibodies, Centrin 20H5 and Centrin 3, as indicated. Whereas each spermatid typically contains a single pair of centrioles (Fig. 4A, white arrowheads), strikingly, large clusters comprising as many as 23 centrioles per cell were detected in cKO testes (Fig. 4B). In the cKO testis, centriole clusters were evident as early as postnatal day 22 (P22) (Fig. 4C, arrowhead), shortly after the appearance of round spermatids during the synchronized first wave of spermatogenesis (Bellvé et al, 1977; Ernst et al, 2019). Analysis by transmission electron microscopy (TEM) revealed that, in control step 2–3 spermatids, basal bodies were properly docked to the plasma membrane and associated with flagella, whereas cKO step 8 spermatids exhibited basal body docking defects and contained at least three centrioles (Figs. 4D and EV2). Notably, these centrioles failed to dock to the nuclear envelope but developed a structure resembling the basal plate (Fig. 4D, black arrowhead), which is part of the head-tail coupling apparatus (HTCA) that normally forms between the proximal centriole and nuclear membrane (Buglak et al, 2025; Wu et al, 2020). In addition, manchette microtubule bundles were visible in the lower cKO image (Fig. 4D, red arrowheads). To assess whether centriole clusters in cKO testes preserve the normal 1:1 ratio of mother to daughter centrioles, we performed IF staining of adult testis sections using antibodies against centrin and ODF2 (a mother centriole marker). As shown in Fig. 4E, centriole clusters in cKO testes retained the expected mother-to-daughter ratio. A single ODF2-positive mother centriole was detected in 100% (50/50 centriole pairs) of control round spermatids and in 98% (57/58 centriole pairs) of cKO round spermatids. Together, these data suggest that loss of CEP164 results in basal body docking defects and abnormal centriole aggregation in spermatids while maintaining centriole identity.

### Centriole cluster fate, manchette dynamics, and sperm tail component mislocalization in cKO spermatids

**Figure 5:**
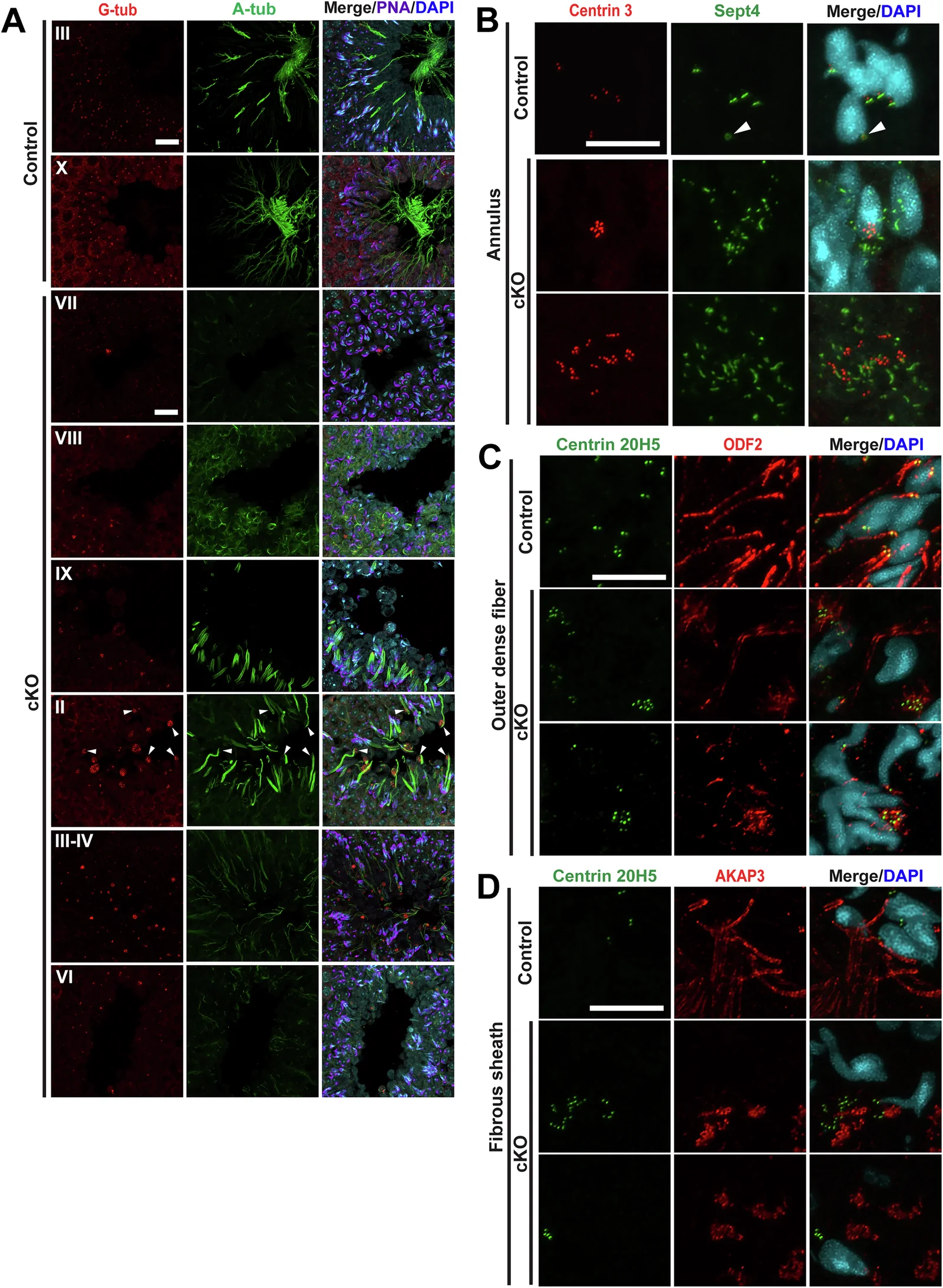
Centriole cluster fate, manchette dynamics, and altered localization of sperm tail structural components in cKO mice. (**A**) IF staining of adult testis paraffin sections for G-tub and A-tub, counterstained with PNA and DAPI. Spermatogenic stages are indicated in the upper left corner. Arrowheads denote centriole clusters associated with the distal tip of manchettes. Scale bars, 20 µm. (**B**–**D**) IF staining of adult testis frozen sections using the following antibody combinations: (**B**) Centrin 3 and Sept4 (annulus component), (**C**) Centrin 20H5 and ODF2 (outer dense fiber component), and (**D**) Centrin 20H5 and AKAP3 (fibrous sheath component). Scale bars, 10 µm. In (**B**), note that most Sept4-positive annuli in control samples are shown in side view, with one example showing a ring-like structure (arrowhead). In addition, the bottom cKO images were acquired in the seminiferous tubule lumen, where nuclei were absent.

To track the fate of centriole clusters across different stages of spermatogenesis in cKO spermatids, we performed IF staining of adult testis sections using antibodies against A-tub and G-tub, with PNA and DAPI counterstaining (Fig. 5A). In control seminiferous tubules, flagella were clearly labeled with the A-tub antibody, whereas the manchette was barely detectable in paraffin sections following citrate antigen retrieval. In contrast, in cKO testes, the manchette was readily detectable with the A-tub antibody, suggesting that α-tubulin in cKO manchettes is highly acetylated. Manchette formation in cKO spermatids appeared to initiate at step 8 (stage VIII) with normal timing, and from step 9 (stage IX) through early step 14 (stage II), the manchette appeared substantially elongated. Intriguingly, centriole clusters were frequently observed at the distal tips of manchettes in the lumen (Fig. 5A, arrowheads), suggesting that these centriole clusters may be subsequently discarded into residual bodies. Notably, in late step 14 to early step 15 spermatids (stage III–IV), numerous centriole clusters were detected in the lumen, and A-tub-positive manchettes displayed a marked reduction in intensity, consistent with disassembly. A gradual decrease in manchette staining intensity was observed from stages III to VI, and only residual staining was detected in step 15 spermatids (stage VI).

**Figure EV3:**
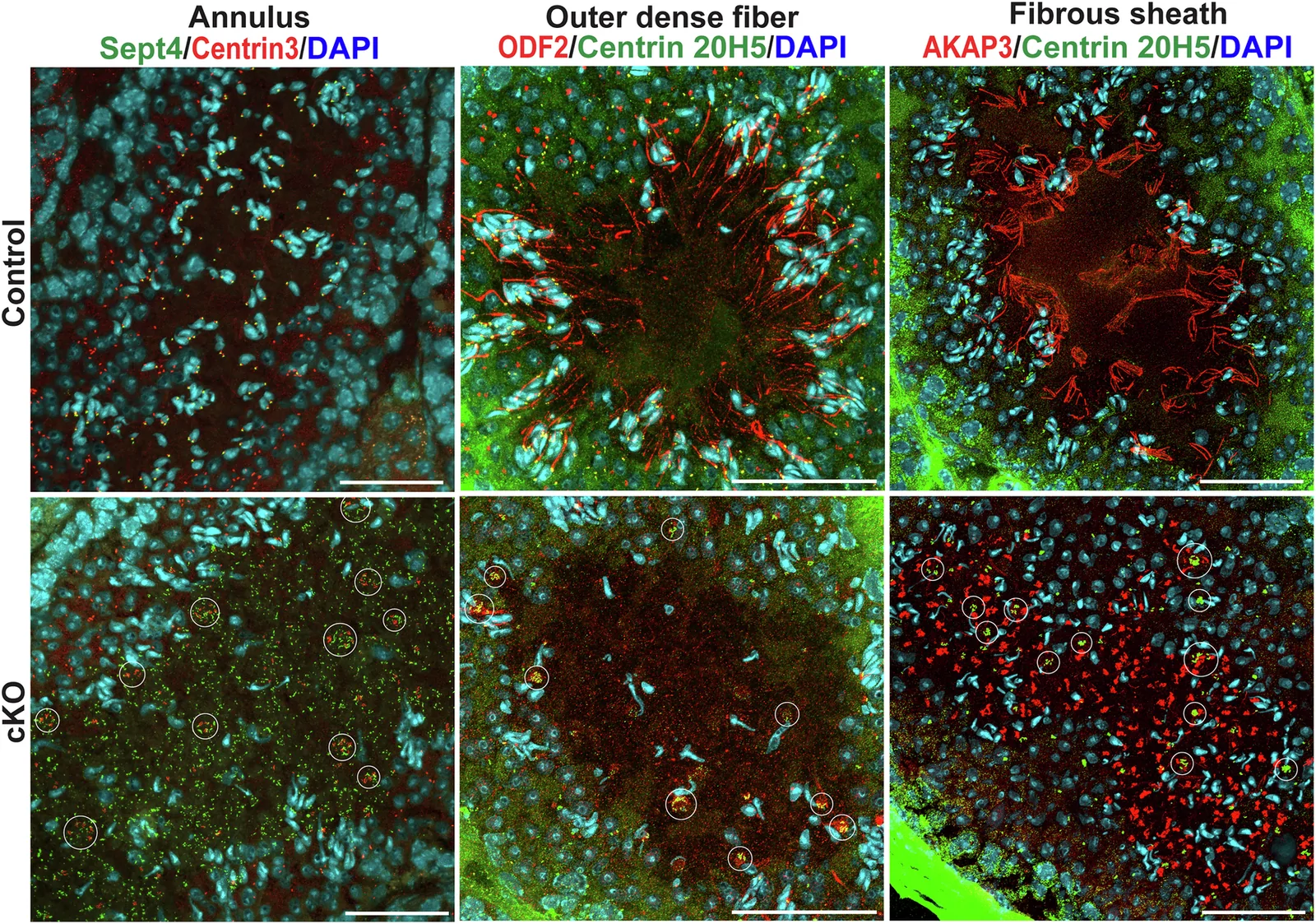
IF analysis of sperm tail structural components in adult testes. Adult testis frozen sections were immunostained using the following antibody combinations: Centrin 3 and Sept4 (annulus component), Centrin 20H5 and ODF2 (outer dense fiber component), and Centrin 20H5 and AKAP3 (fibrous sheath component) as indicated. Centriole clusters are outlined by circles. Scale bars, 50 µm.

We next investigated the localization of structural components of the sperm tail. IF staining of adult testis sections was performed using antibodies against centrin and specific sperm tail markers. Sept4 is a major component of the annulus that is present as a distinct ring at the base of the flagellum distal to the centrin-positive basal body during spermiogenesis steps 9–14 (Hoque et al, 2024; Whitfield, 2024). Whereas such Sept4 rings were readily detectable at the flagellar base in controls, no clear Sept4 rings were observed in cKO spermatids (Fig. 5B). Instead, elongated Sept4-positive fibrous structures were frequently noted. ODF2, an outer dense fiber protein of the midpiece and principal piece, and AKAP3, a fibrous sheath component of the principal piece, also displayed abnormal distribution in cKO spermatids. Rather than outlining axonemal structures as in controls (Fig. 5C,D), both proteins accumulated around aggregated centrioles. Moreover, centriole clusters, together with mislocalized sperm tail components, were abundant in the lumen of seminiferous tubules (Fig. EV3), suggesting that these aberrant structures are discarded through residual bodies. These findings highlight the critical role of CEP164 in the proper assembly of sperm tail structures.

### Centriole clusters first emerge in round spermatids of cKO testes

**Figure 6:**
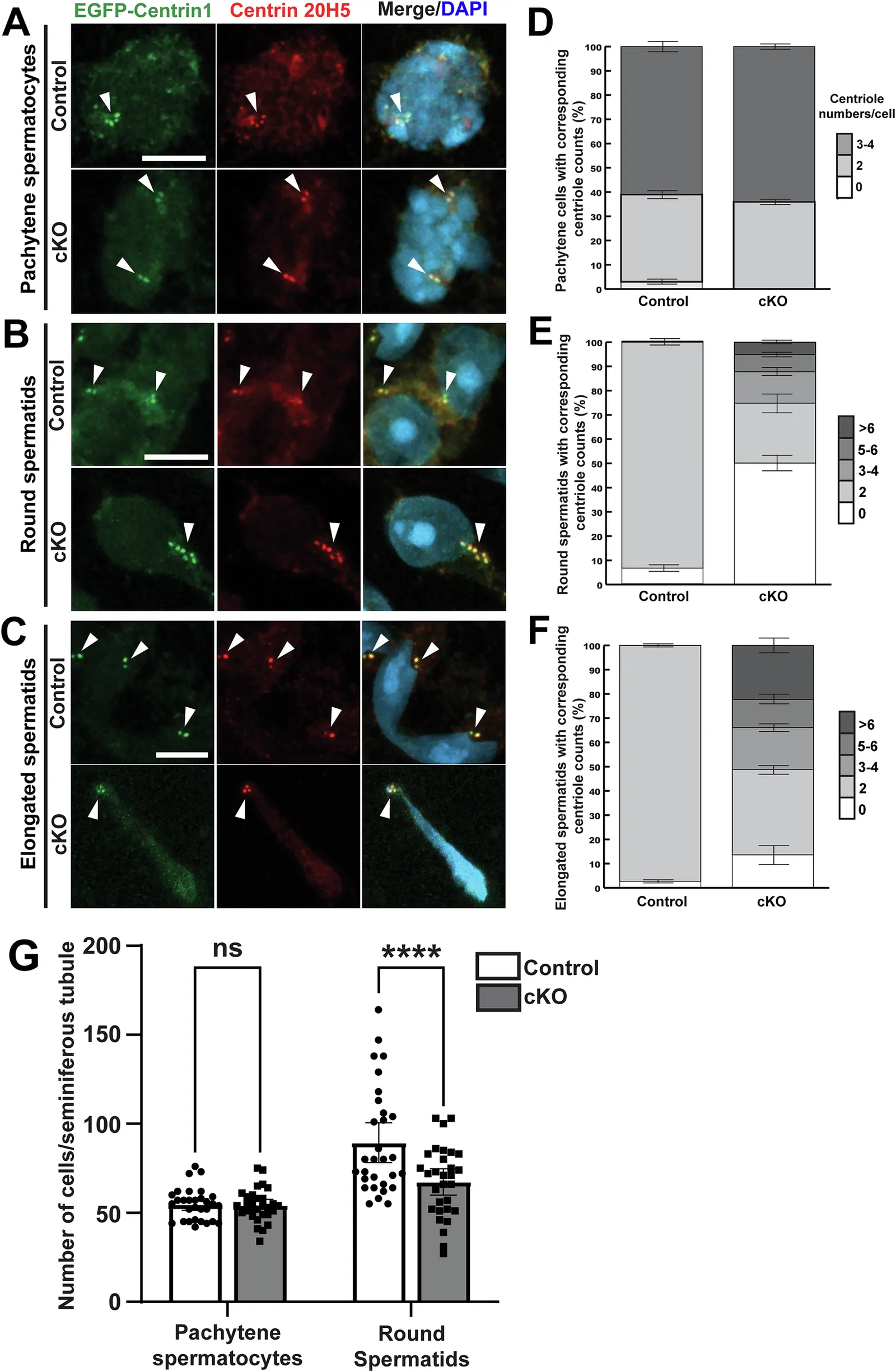
Centriole clusters emerge at the round spermatid stage. (**A**–**C**) IF staining of seminiferous tubule squashes from mice expressing EGFP-Centrin1 using Centrin 20H5 antibody. Representative images of (**A**) pachytene spermatocytes, (**B**) round spermatids, and (**C**) elongated spermatids are shown. Arrowheads point to centrioles. Scale bars, 5 µm. (**D**–**F**) Quantification of centriole counts in the corresponding cell types. *n* = 5 control and *n* = 3 cKO mice, with 50 pachytene spermatocytes quantified per mouse. *n* = 5 control and *n* = 3 cKO mice, with 100 round spermatids quantified per mouse. *n* = 4 control and *n* = 3 cKO mice, with 100 elongated spermatids quantified per mouse. Error bars represent ± SEM. (**G**) Quantification of pachytene spermatocytes and round spermatids in cross-sections of control and cKO postnatal testes (P25-26), immunostained for SYCP1 and SYCP3. *n* = 3 mice per genotype, with 10 seminiferous tubules analyzed per mouse. Error bars represent ± SEM. Šidák multiple comparisons test: Pachytene spermatocytes, not significant (ns), *P* = 0.9944; Round spermatids, ****P = 0.000036. Source data are available online for this figure.

To determine when centriole clustering first arises in cKO testes, we prepared seminiferous tubule squashes from mice expressing EGFP-Centrin1 and performed IF staining using Centrin 20H5 antibody for precise centriole detection. Quantification across spermatogenic stages revealed that the majority of pachytene spermatocytes in both control and cKO testes typically contained four centrioles (Fig. 6A,D) as reported (Ho et al, 2021; Marjanovic et al, 2015). In contrast, while most control round spermatids contained two centrioles, 25.2% of cKO round spermatids displayed more than two, and surprisingly, 50.1% lost centrioles entirely (Fig. 6B,E). This pattern persisted in elongated spermatids, which showed an increased frequency of cells with supernumerary centrioles (Fig. 6C,F). However, the proportion of acentriolar elongated spermatids was markedly reduced, suggesting that round spermatids devoid of centrioles are eliminated during spermatogenesis. Collectively, we conclude that centriole clustering first emerges at the round spermatid stage in the absence of CEP164.

**Figure EV4:**
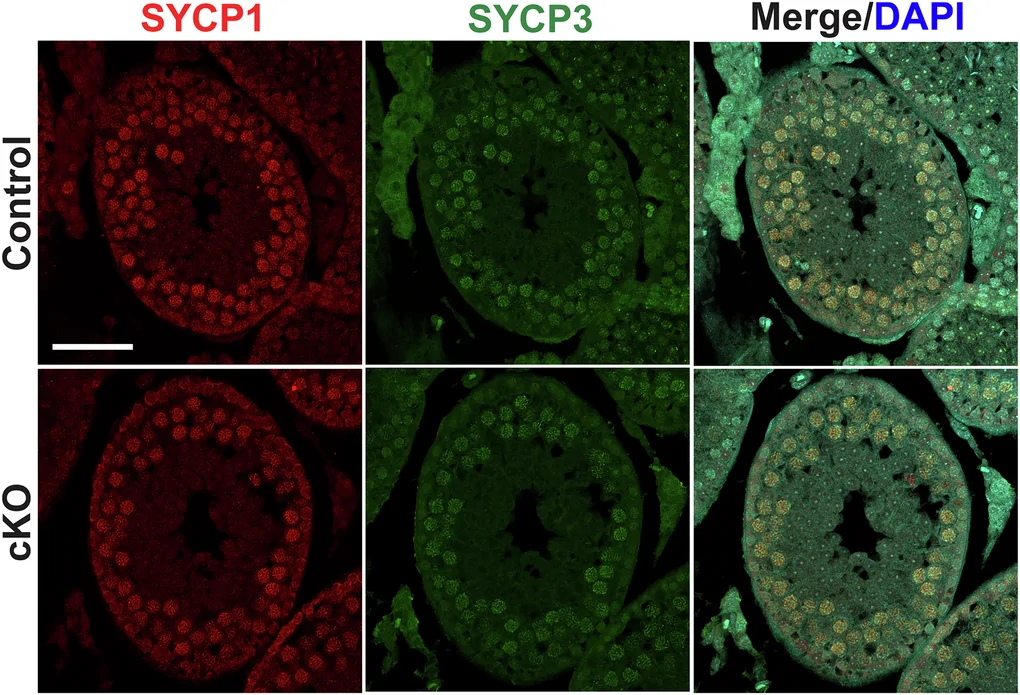
Representative IF images of P25 testis sections immunostained for SYCP1 and SYCP3. Testis paraffin sections from P25 littermates during the synchronized first wave of spermatogenesis were immunostained for SYCP1 and SYCP3. Scale bars, 50 µm.

To further substantiate the reduction in round spermatids observed in the cKO testis, we quantified germ cell populations during the synchronized first wave of spermatogenesis by IF staining of postnatal testes (P25 and P26) for SYCP1 and SYCP3 (Fig. EV4). Quantification revealed no significant difference in the number of pachytene spermatocytes between control and cKO testes (Fig. 6G). However, the number of round spermatids was significantly reduced in the cKO testis. As no morphological signs of apoptosis were observed, and neither cleaved caspase-3 immunostaining nor TUNEL assays detected apoptotic cells, we speculate that abnormal spermatids in the cKO testis may instead be eliminated through alternative mechanisms, such as Sertoli cell-mediated phagocytosis or exfoliation into the seminiferous tubule lumen (Franca et al, 2016; O’Donnell et al, 2022; San Agustin et al, 2015).

### Centriole pairs are highly mobile and cluster together in cKO round spermatids

**Figure 7:**
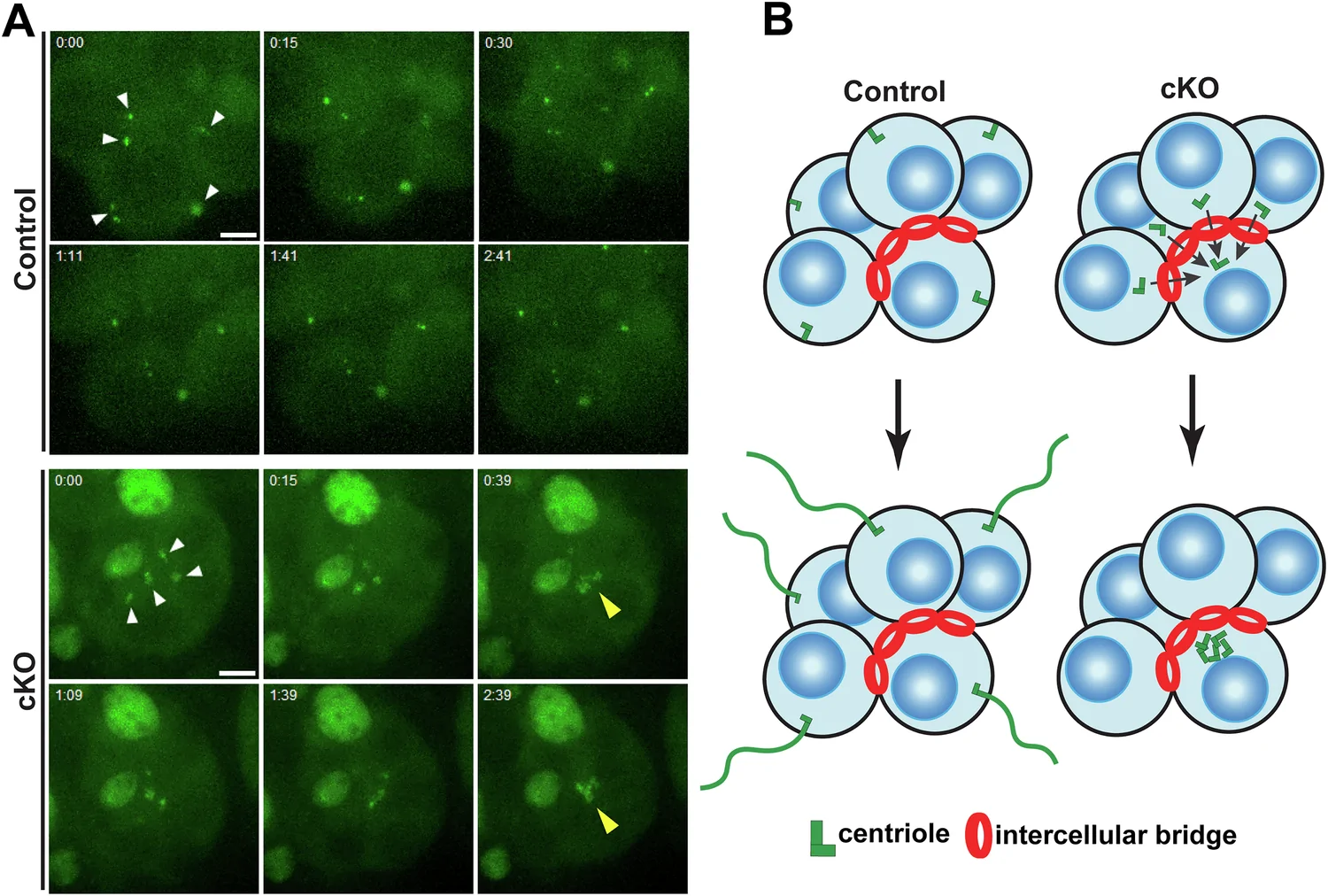
Live-cell imaging of centrioles in round spermatids. (**A**) Representative spinning disk confocal time-lapse images of EGFP-Centrin1 in round spermatids. Time points are displayed in the upper left corner (h:mm). White arrowheads mark the initial positions of centriole pairs, while yellow arrowheads highlight centriole aggregates in cKO round spermatids. Large round green structures in the cKO sample correspond to autofluorescent floating cells. Scale bars, 5 µm. (**B**) Schematic model illustrating a hypothetical mechanism for centriole clustering in cKO round spermatids. Defective basal body docking renders centrioles mobile, allowing them to interact via intercellular bridges, ultimately forming large aggregates.

We considered two possible mechanisms for the formation of centriole clusters: (1) de novo centriole amplification, as reported in cancer cells (Kiermaier et al, 2024; Mittal et al, 2021) or spermatocytes with dysregulation of polo-like kinases (Skinner et al, 2024; Wellard et al, 2021), and (2) centriole migration, similar to centrosome migration through nurse cells into oocytes in *D*. *melanogaster* (Bonente et al, 2025; Hannaford and Rusan, 2024). To distinguish between these possibilities, we performed live-cell imaging of centrioles at 15-min intervals in isolated round spermatids from mice expressing EGFP-Centrin1. In control spermatids, centrioles appeared stably anchored to the plasma membrane, and centriole pairs remained distant from one another (Fig. 7A; Movie EV1). In striking contrast, centrioles in cKO spermatids are frequently associated with one another, likely through intercellular bridges, to form aggregates (Fig. 7A, yellow arrowheads; Movie EV2). No evidence of centriole amplification was detected over three independent 24-h live-cell recording sessions. To more precisely examine centriole dynamics in cKO spermatids at higher temporal resolution, we performed live-cell imaging at 16 s intervals. Consistent with the above results, cKO centrioles were highly mobile and associated with one another (Movie EV4), while control centriole pairs were largely stationary (Movie EV3). These observations suggest that loss of CEP164 and defective basal body docking render centrioles highly mobile, potentially allowing them to pass through intercellular bridges and cluster together, thereby leading to centriole aggregation (Fig. 7B).

## Discussion

The distal appendage protein CEP164 plays a pivotal role in basal body docking and ciliogenesis in somatic cells. However, its function during germ cell differentiation in mammals has remained largely unknown. In the seminiferous tubules of the adult murine testis, at least two types of cilia have been described: zygotene cilia and sperm flagella (Lopez-Jimenez et al, 2022). Although the role of sperm flagella in driving motility and fertilization is well established, the biological significance of zygotene cilia in the testis is not yet understood.

Here, we conditionally ablated CEP164 in male germ cells using the *Stra8*-iCre driver line. In the resulting cKO testis, sperm flagella and zygotene cilia were nearly absent (Figs. 2A,C and 3A,B). In striking contrast to zebrafish oogenesis, where zygotene cilia are essential for meiosis-specific chromosomal events (Mytlis et al, 2022; Mytlis et al, 2023), no major lesions were apparent in chromosome pairing and DSB repair in the absence of CEP164 (Fig. 3C–H). Although we can not formally exclude the possibility that a small number of residual zygotene cilia may be sufficient to support these processes, potentially through syncytial communication between germ cells connected by intercellular bridges, our findings nevertheless suggest that zygotene cilia are dispensable for the proper progression of male meiosis in mice. This observation is in agreement with previous reports showing that testis-specific *Kif3A*-KO and *Ift88* hypomorphic mice, deficient in key components of the intraflagellar transport (IFT) machinery essential for ciliogenesis, undergo largely normal spermatogenesis up to the round spermatid stage but subsequently develop pronounced defects in flagellar assembly (Lehti et al, 2013; San Agustin et al, 2015). However, the status of zygotene cilia and meiotic events was not examined in these studies. Furthermore, a recent study reported that only ~20% of zygotene spermatocytes possess primary cilia, and that these cilia are not directly associated with bouquet formation during early meiosis (Lopez-Jimenez et al, 2022). The mechanistic basis for why only a subset of mouse zygotene spermatocytes forms primary cilia remains unclear, particularly when contrasted with zebrafish oocytes, where the majority of zygotene cells are ciliated (Mytlis et al, 2022). This difference may reflect sex-specific or species-dependent regulatory mechanisms. In mice, it is conceivable that ciliated zygotene spermatocytes serve as specialized sensory or signaling centers that relay regulatory information to adjacent non-ciliated germ cells within the syncytium. Given the extensive intercellular continuity mediated by cytoplasmic bridges, such a mechanism could promote synchronized progression of germ cell differentiation. Collectively, our findings suggest that the male infertility observed in cKO mice arises from defects in basal body docking and flagellogenesis during spermiogenesis, rather than abnormalities in meiosis. Interestingly, unlike in mice, germ cell-specific deletion of ciliary genes in zebrafish disrupts meiotic progression by impairing DSB repair and crossover formation, leading to increased germ cell apoptosis and male infertility (Liu et al, 2023; Xie et al, 2022). This points to an evolutionarily divergent requirement for spermatocyte cilia in regulating gametogenesis across vertebrates.

What is the potential role of zygotene cilia in the mammalian testis? One possibility is that zygotene cilia may function as sensory organelles that regulate spermatogenesis under physiological or environmental stress conditions such as starvation, inflammation, or exposure to chemical or hormonal cues, all of which are known to influence sperm production (Fanjul and Ruiz de Galarreta, 1981; Hasan et al, 2022; Sharpe, 2010; Sofikitis et al, 2008). Another potential role may involve the blood-testis barrier (BTB), a tight junctional barrier formed by adjacent Sertoli cells that divides seminiferous tubules into the basal compartment and apical (adluminal) compartments. (Luaces et al, 2023; Mruk and Cheng, 2015). As preleptotene/leptotene spermatocytes traverse the BTB into a new adluminal environment, zygotene cilia may serve as environmental sensors that monitor local stimuli to modulate germ cell differentiation under certain circumstances. Clearly, further investigation is required to elucidate the precise functions of zygotene cilia during spermatogenesis.

The manchette is a transient, microtubule-based structure that assembles around the nucleus of elongating spermatids during mammalian spermiogenesis (Gao et al, 2025; Lehti and Sironen, 2016). The manchette functions as a specialized cytoskeletal platform that facilitates nuclear shaping, acrosome maturation, and flagellar assembly. Perturbations of manchette organization are frequently associated with sperm head malformation, teratozoospermia, and male infertility. In mice, the manchette first becomes evident in step 8 spermatids, undergoes progressive elongation until around step 12, and is subsequently dismantled during steps 13-14, coincident with the completion of nuclear morphogenesis. In the cKO testes, we observed a pronounced hyper-elongation of the manchette structure (Figs. 2C–E and 5A). Similar aberrantly extended manchettes have been reported in mouse models with defects in IFT or ciliogenesis, including mutants of *Kif3a*, *Ift88*, and *Ift20* (Gao et al, 2025; Lehti et al, 2013; San Agustin et al, 2015; Zhang et al, 2016). Notably, the manchette microtubules in cKO spermatids exhibited marked hyperacetylation of α-tubulin, a post-translational modification typically enriched in stable axonemal microtubules of cilia and flagella. We speculate that impaired flagellogenesis alters intracellular tubulin partitioning, resulting in tubulin subunits normally destined for axonemal assembly being redirected toward manchette microtubules. Despite these structural abnormalities, the overall timing of manchette assembly and disassembly did not appear substantially altered, raising the possibility that the manchette in cKO spermatids may assemble more rapidly or continue to elongate until near the onset of disassembly.

In *Drosophila melanogaster*, the CEP164 ortholog is not essential for basal body docking, flagellogenesis, and male fertility (Hou et al, 2023). In contrast, our findings demonstrate that CEP164 is indispensable for male fertility in mice. In the absence of CEP164, spermatogenesis proceeds normally up to the round spermatid stage. However, round spermatids exhibit impaired basal body docking and defective flagellogenesis. Intriguingly, undocked centrioles are highly dynamic and mobile, often associating with one another to form aggregates (Fig. 7B). One possible explanation is that these interactions occur through intercellular bridges connecting spermatids, which typically measure ~0.5–3.0 μm in diameter and could theoretically permit centriole passage (Greenbaum et al, 2011). Consistent with this idea, we observed a concomitant increase in acentriolar spermatids. However, due to technical limitations of our live-cell imaging approach, plasma membranes and intercellular bridges could not be directly visualized, and therefore it remains unclear whether centrioles physically transit through intercellular bridges. Centriole clusters are frequently observed in the lumen of seminiferous tubules, suggesting that they are disposed of within residual bodies (Fig. 5A and EV3). Centriole clustering in the cKO testis is reminiscent of centriole migration into the oocyte in *Drosophila melanogaster* (Bonente et al, 2025; Hannaford and Rusan, 2024). In most metazoans, centrioles are eliminated during oogenesis. In early *Drosophila* oogenesis, a cyst of 16 germ cells interconnected by cytoplasmic bridges forms, and centrioles from the 15 nurse cells migrate through these bridges into the designated oocyte, where they are ultimately discarded as the oocyte matures. Recent evidence indicates that centriole migration through the germline cytoplasm is an evolutionarily conserved process that also occurs in mice (Lei and Spradling, 2016; Niu and Spradling, 2022). One intriguing possibility is that CEP164 and its associated centriolar distal appendage structures may be dispensable for oogenesis. We were unable to directly test this hypothesis, as *Stra8*-iCre lacks activity in female germ cells (Sadate-Ngatchou et al, 2008). Nonetheless, male germ cells lacking CEP164, with undocked centrioles, appear to adapt a female-like phenotype, characterized by centriole clustering and elimination.

Our study underscores the importance of basal body docking in centriole retention in spermatids, suggesting that basal body docking may regulate biological processes beyond ciliogenesis. Consistent with this notion, unciliated membrane-anchored centrioles have been described in mature cerebellar granule cell (GC) neurons (Ott et al, 2024), cytotoxic T-lymphocytes (CTLs) (Douanne and Griffiths, 2021; Stinchcombe et al, 2006), and zona reticularis cells in the adrenal cortex (Wheatley, 1967). Although the functional significance of docked centrioles in GCs and zona reticularis cells remains poorly defined, in CTLs, the mother centriole transiently docks at the plasma membrane adjacent to target cells, where it orchestrates cytoskeletal remodeling necessary for the polarized secretion of cytotoxic granules at the immunological synapse. Collectively, these observations raise the possibility that unciliated docked centrioles may be more prevalent than previously appreciated and may fulfill active, cilia-independent roles in diverse cellular contexts.

Aberrant centriole clusters are frequently observed in aggressive human cancers and are known to promote multipolar spindle formation, chromosomal instability, and aneuploidy (Kiermaier et al, 2024; Mittal et al, 2021). The presence of supernumerary centrioles correlates with enhanced metastatic potential and poor clinical prognosis (Kiermaier et al, 2024; LoMastro and Holland, 2019). Although centriole amplification is widely regarded as the principal cause of increased centriole numbers, centriole migration through intercellular bridges may present a novel mechanism. The occurrence and function of intercellular bridges in cancer cells have not been thoroughly investigated. However, intercellular bridges or tunneling nanotubes as wide as ~5 μm in diameter have been reported in cancer cells (Asencio-Barria et al, 2019; Vidulescu et al, 2004), providing sufficient space for centrioles to travel between connected cells.

In summary, our findings demonstrate that zygotene cilia are dispensable for the proper progression of male meiosis in mice. The distal appendage protein CEP164 is essential for basal body docking, centriole immobilization and retention, and flagellogenesis during murine spermatogenesis. These results illustrate the evolutionary and biological complexity of gametogenesis, reflecting the presence of species- and sex-specific adaptations in primary cilia function.

## Methods

**Table:** Reagents and Tools Table

Reagent/resource	Reference or source	Identifier or catalog number
**Experimental models**		
*Stra8*-iCre	Jackson Lab	RRID:IMSR_JAX:008208
*Cep164* ^*fl/fl*^	Siller et al, 2017	
EGFP-Centrin1	Hirai et al, 2016	
*Stra8-iCre;Cep164* ^*fl/fl*^ *;EGFP-Centrin1*	N/A	
*Stra8-iCre;Cep164* ^*fl/fl*^	N/A	
**Recombinant DNA**		
**Antibodies**		
Acetylated α-tubulin [6-11B-1]	Sigma-Aldrich	AB_477585
Alpha tubulin	Sigma	AB_477583
AKAP3	Proteintech	AB_2273887
Centrin [20H5]	Sigma-Aldrich	AB_10563501
Centrin 3 [3E6]	Abnova	AB_537701
CEP164 [17]	Sigma-Aldrich	SAB2702371
Gamma tubulin, [GTU-88]	Sigma	AB_477584
ODF2	Proteintech	AB_2156630
Phospho-H2A.X (Ser139)	Sigma-Aldrich	AB_11213838
Phospho-Histone H3 (Ser10)	Sigma-Aldrich	AB_310177
Rad51	Sigma-Aldrich	AB_2238184
SEPT4	Proteintech	AB_2187151
SYCP1	Invitrogen	AB_2302734
SYCP3 [D-1]	Santa Cruz	AB_2197353
Anti-Mouse IgG Alexa Fluor 488 PLUS	Invitrogen	AB_2633275
Anti-Mouse IgG1 Alexa Fluor 488	Invitrogen	AB_2535764
Anti-Mouse IgG2a DyLight 488	Jackson ImmunoResearch Labs	115-485-206
Anti-Mouse Alexa Fluor 555 PLUS	Invitrogen	AB_2633276
Anti-Mouse Alexa Fluor 594 PLUS	Invitrogen	AB_2762825
Anti-Mouse IgG1 ML DL549	Jackson ImmunoResearch Labs	115-505-205
Anti-Mouse IgG2a DyLight 549	Jackson ImmunoResearch Labs	115-505-206
Anti-Mouse IgG2b DyLight 549	Jackson ImmunoResearch Labs	115-505-207 (91425)
Anti-Mouse IgG1 DyLight 649	Jackson ImmunoResearch Labs	115-495-205
Anti-Rabbit Alexa Fluor 488 PLUS	Invitrogen	AB_2633280
Anti-Rabbit Alexa Fluor 555 PLUS	Invitrogen	AB_2633281
Anti-Rabbit Alexa Fluor 594 PLUS	Invitrogen	AB_2762824
Peanut Agglutinin (PNA), Rhodamine	Vector Labs	RL-1072-5
**Oligonucleotides and other sequence-based reagents**		
**Chemicals, enzymes and other reagents**		
Bovine serum albumin	Sigma-Aldrich	A4503
Paraformaldehyde	Sigma-Aldrich	P6148
Periodic acid-Schiff (PAS) staining system	Sigma-Aldrich	395B-1KT
Bouin’s fixative solution	Ricca Chemical	1120-32
Tween 20	Sigma-Aldrich	P1379
Triton X-100	Sigma-Aldrich	T9284
Xylene	Fisher Scientific	22-110-676
Hematoxylin solution, Gill No. 3	Poly Scientific	S211
Eosin-Phloxine working solution	Poly Scientific	S176
Flouromount-G	Southern Biotech	0100-01
OCT compound	Tissue-Tek	62550-01
PBS	Sigma-Aldrich	P3813
Sucrose	Sigma-Aldrich	G5767
MEMα	Gibco	12571063
Acetone	Fisher Scientific	A18-4
Methanol	Fisher Scientific	A412-4L
Penicillin-streptomycin solution, 100x	Corning	30-002-CI
TEM embedding resin	EMS	14120
Sodium citrate tribasic dihydrate	Sigma-Aldrich	C8532-1KG
KnockOut serum replacement	Gibco	10828028
EDTA	Sigma-Aldrich	E5391
DTT	Sigma-Aldrich	D9779
PMSF	Sigma-Aldrich	P7626
Trizma, hydrochloride	Sigma-Aldrich	T3253
Permount	Fisher Scientific	SP15-100
Goat serum	Gibco	16210072
**Software**		
Fiji ImageJ	https://imagej.net/software/fiji/downloads	
Zeiss Zen lite	https://www.zeiss.com/microscopy/en/products/software/zeiss-zen-lite.html	
NIS-Elements	https://www.microscope.healthcare.nikon.com/products/software/nis-elements	
Leica Application Suite X	https://www.leica-microsystems.com/products/microscope-software/p/leica-las-x-ls/	
Adobe Suite	https://www.adobe.com/creativecloud.html	
GraphPad	https://www.graphpad.com/	
**Other**		
Charged microscope slides	Fisher Scientific	22230890

### Mouse lines

All animal procedures were conducted in accordance with NIH guidelines and approved by the Institutional Animal Care and Use Committee (IACUC) of Stony Brook University (protocol #245427). The generation and genotyping of *Cep164*^*fl/fl*^ mice have been described previously (Siller et al, 2017). *Stra8*-iCre mice were obtained from the Jackson Laboratory (RRID:IMSR_JAX:008208). Mice homozygous for the *Cep164* flox allele and hemizygous for the *Stra8*-iCre transgene (*Stra8-iCre;Cep164*^*fl/fl*^, cKO) were generated by breeding *Stra8-iCre;Cep164*^*fl/+*^ mice with *Cep164*^*fl/fl*^ mice. Adult mice (2–5 months of age) were used for all the experiments unless otherwise specified. *Cep164*^*fl/fl*^ mice were used as controls throughout the study, except for experiments with postnatal mice in Figs. 4C,D, 6G, EV1D,E, and EV4. For these experiments, age-matched littermates were used as controls to minimize experimental variability. Control genotypes included *Cep164*^*fl/fl*^, *Cep164*^*fl/+*^, and *Stra8-iCre;Cep164*^*fl/+*^. For the quantification shown in Fig. 6G, one P25 and two P26 cKO mice were analyzed. For the quantification in Fig. EV1E, one P20, one P22, and one P23 cKO mice were analyzed. In each case, animals were analyzed together with their respective littermate controls. Data from these animals were pooled and plotted in Figs. 6G and EV1E. For live-cell imaging of centriole behavior in spermatids, *Stra8-iCre;Cep164*^*fl/+*^ mice were crossed with *Cep164*^*fl/fl*^*;EGFP-Centrin1* mice to obtain *Stra8-iCre;Cep164*^*fl/fl*^*;EGFP-Centrin1* experimental mice and control *Cep164*^*fl/fl*^*;EGFP-Centrin1* control mice. The *Stra8*-iCre and EGFP-Centrin1 transgenes were maintained in the hemizygous state. Animals were maintained under pathogen-free conditions on a 12:12 h light:dark cycle with free access to food and water in the Division of Laboratory Animal Resources at Stony Brook University.

### Quantification of cauda epididymal sperm

Cauda epididymal sperm were isolated and counted as previously described (Hoque et al, 2024). Briefly, 2–5-month-old mice were euthanized, and a single cauda epididymis was dissected and minced in a 35-mm Petri dish containing 1 ml PBS (pH 7.4). The tissue was incubated at 37 °C for 15 min to allow sperm release. The suspension was collected, centrifuged, and the sperm-containing pellet was resuspended in 1 ml PBS. Sperm were counted using a hemocytometer with appropriate dilutions.

### Histological staining

Testes and epididymides were collected from 2 to 3-month-old mice, fixed overnight in Bouin’s solution (Ricca Chemical), and embedded in paraffin. Sections of 5–10 µm were cut, stained with hematoxylin and eosin (H&E; Poly Scientific R&D Corp.) or Periodic Acid-Schiff Kit (PAS; Sigma-Aldrich), and mounted with Permount (Fischer Scientific).

### Immunofluorescence staining

For immunofluorescence (IF) staining of paraffin-embedded tissues, samples were fixed in 4% paraformaldehyde, paraffin-embedded, and sectioned. Sections were deparaffinized, rehydrated, and subjected to antigen retrieval in sodium citrate buffer (10 mM sodium citrate, 0.05% Tween 20, pH 6.0) using an autoclave for 17.5 min. Sections were permeabilized with 0.5% Triton X-100 in PBS for 15 min, washed three times with 0.05% Tween 20 in PBS (5 min each), and blocked for 1 h at room temperature in 5% goat serum prepared in antibody dilution solution (2% BSA in PBS). Primary antibodies were applied in antibody dilution buffer and incubated overnight at 4 °C. After three washes with 0.05% Tween 20 in PBS, sections were incubated with secondary antibodies for 1 h at room temperature, washed three additional times with 0.05% Tween 20 in PBS, counterstained with DAPI for 2 min, and mounted with Fluoromount-G (Southern Biotech).

For IF staining of frozen tissues, freshly isolated testes were embedded in OCT compound (Tissue Tek), frozen at −80 °C overnight, and sectioned at 5–10-µm thickness. Sections were air-dried for 15 min and fixed in cold methanol:acetone (1:1) for 10 min. Subsequent steps were performed as described above, except the Triton X-100 permeabilization step was omitted.

### Seminiferous tubule squash and meiotic chromosome spread preparations

Seminiferous tubule squashes were performed as previously described (Wellard et al, 2018) with modifications. Testes from 2 to 4-month-old mice were harvested, detunicated, and individual seminiferous tubules were incubated in fix/lysis solution (0.8% PFA and 0.1% Triton X-100 in PBS) for 5 min. Seminiferous tubules were then cut into 5–20 mm fragments and transferred onto charged slides pre-coated with fix/lysis solution. Coverslips were placed over the tubules, and pressure was applied with the heel of the palm for 30 s. Slides were immediately flash-frozen in liquid nitrogen for ~20 s and used immediately for IF staining or stored at −80 °C. For IF staining, slides were reimmersed in liquid nitrogen, and coverslips were removed with a razor blade.

Meiotic chromosome spreads were prepared as previously described (Dia et al, 2017) with modifications. Testes from 2 to 3-month-old mice were harvested, detunicated, and incubated in hypotonic extraction buffer (HEB; 30 mM Tris-HCl pH 8.2, 50 mM sucrose, 17 mM trisodium citrate dihydrate, 5 mM EDTA, 0.5 mM DTT, and 0.1 mM PMSF) for 1 h. Seminiferous tubule clumps (~ 3 mm in diameter) were then minced in 100 mM sucrose solution and spread onto slides pre-coated with fixative solution (1% PFA, 0.15% Triton X-100). Slides were dried in a covered humidified chamber for 3 h, followed by 30 min in an open humidified chamber. After three 5-min PBS washes, slides were air-dried vertically for 20 min and either used immediately for IF staining or stored at −80 °C.

### Transmission electron microscopy

TEM was performed as described previously (Hoque et al, 2024; Siller et al, 2017). Testes from P26 mice were dissected, cut into ~1 mm^3^ pieces, and fixed overnight at 4 °C in 4% PFA and 2% glutaraldehyde in PBS. Samples were rinsed several times with PBS, post-fixed with 2% osmium tetroxide for 1 h, dehydrated through a graded ethanol series, followed by acetonitrile, and embedded in Embed 812 resin (Electron Microscopy Sciences) for 48 h at 60 °C. Ultrathin sections (60–90 nm) were cut on a Leica EM UC7 ultramicrotome, collected on Formvar-coated slot copper grids, and counterstained with 1% methanolic uranyl acetate and 0.3% aqueous lead citrate. Images were acquired on a FEI Tecnai G2 12 BioTwin TEM equipped with an XR-60 CCD digital camera system (Advanced Microscopy Techniques).

### Live-cell imaging of centriole dynamics in spermatids

Testes were collected from 2 to 3-month-old mice expressing EGFP-Centrin1, detunicated, and seminiferous tubules were transferred to a sterile Petri dish containing PBS. Tubule segments at spermatogenic stages I–V, which contain round spermatids, were identified by phase-contrast microscopy using the transillumination method (Kotaja et al, 2004). Selected segments were isolated and transferred to 35-mm culture dishes containing MEMα (Gibco) supplemented with 10% KnockOut Serum Replacement (KSR; Gibco) and 100 U/mL penicillin–streptomycin. Tubules were gently minced to release clumps of spermatids, which were then allowed to disperse. Live-cell imaging was performed using a Nikon Eclipse Ti2-E CrestOptics X-Light V3 spinning disk confocal system equipped with a 40×/0.90 NA objective and an environmental chamber at 37 °C and 5% CO_2_. Images were acquired at either 15-min intervals for 24 h or 16-s intervals for 15 min.

### Image acquisition and analysis

For histological staining, images were acquired using a Leica DMI6000B microscope equipped with either a DFC7000T camera and either a 40×/0.75 NA or a 100x/1.30 NA oil-immersion objective. IF images were obtained using a Zeiss LSM 980 Airyscan 2 confocal microscope with a 100×/1.40 NA oil-immersion objective or a Nikon AXR confocal microscope with either a 20×/0.80 NA or 60×/1.42 NA oil-immersion objective in Galvano scan mode. Image analysis was performed using Leica Application Suite X, Zeiss ZEN lite, or Nikon NIS-Elements software. Deconvolution was performed using the Richardson–Lucy algorithm for images acquired on the Nikon AXR. Airyscan processing was applied to images acquired on the Zeiss LSM 980. Final images were generated using ImageJ, Adobe Photoshop, and Adobe Illustrator.

### Statistical analysis

Statistical analyses involving multiple independent variables were performed using a Šidák multiple comparisons test. In all other cases, two-tailed Welch’s *t* tests were used to determine statistical significance. *P* < 0.05 was considered to be significant. Asterisks were used to indicate *P* values as follows: **P < 0.01; ***P < 0.001; ****P < 0.0001 and ns, not significant. Blinding was not performed unless otherwise stated.

## Supplementary information

Peer Review File

Movie EV1

Movie EV2

Movie EV3

Movie EV4

Source data Fig. 1

Source data Fig. 2

Source data Fig. 3

Source data Fig. 4

Source data Fig. 6

EV Figures Source Data

Expanded View Figures

## Data Availability

The source data of this paper are collected in the following database record: https://www.ebi.ac.uk/biostudies/bioimages/studies/S-BIAD3488. The source data of this paper are collected in the following database record: biostudies:S-SCDT-10_1038-S44319-026-00872-8.
