# Supplementary material for: Basal body docking failure triggers centriole clustering and elimination in mammalian spermatogenesis
Source: EMBO Rep. 2026 Jul 18;27(17):5039–61. doi: 10.1038/s44319-026-00872-8 (PMC13554106; doi:10.1038/s44319-026-00872-8)
Supplement: Supplementary file 1 [file 44319_2026_872_MOESM12_ESM.pdf]

Expanded View Figures

Figure EV1. Generation of *Stra8-iCre;Cep164$^{fl/fl}$* mice.

(**A**) Schematic of the CEP164 protein domain organization, showing a WW domain and three coiled-coil motifs (CCI-CCIII). The arrow marks the truncation site at amino acid 65 following Cre-mediated recombination. (**B**) Strategy for generating *Stra8-iCre;Cep164$^{fl/fl}$* mice. The locations of genotyping primers are indicated by green arrows (P1 and P2). (**C**) PCR-based mouse genotyping. (**D**) IF staining of P23 testis frozen sections using CEP164 and Centrin 20H5 antibodies. Nuclei were visualized by DAPI. Scale bars, 10 and 1 µm (insets). (**E**) Quantification of centriole pairs with CEP164 in round spermatids from control and cKO postnatal testes. $n = 3$ mice per genotype (one littermate pair each at P20, P22, and P23), with over 100 round spermatids counted per mice. Round spermatids are abundantly present at P20-P23 during the first wave of spermatogenesis. Error bars represent ± SEM. Welch's *t* test: $^{**}P = 0.0035$. (**F**) IF staining of adult testis paraffin sections using phospho-histone H3 (Ser10) antibody, counterstained with PNA and DAPI. Scale bar, 10 µm. (**G**) Quantification of mitotic spermatogonia. $n = 3$ mice per genotype, with 40 tubules counted per mouse. Error bars represent ± SEM. Welch's *t* test: not significant (ns), $P = 0.7596$.

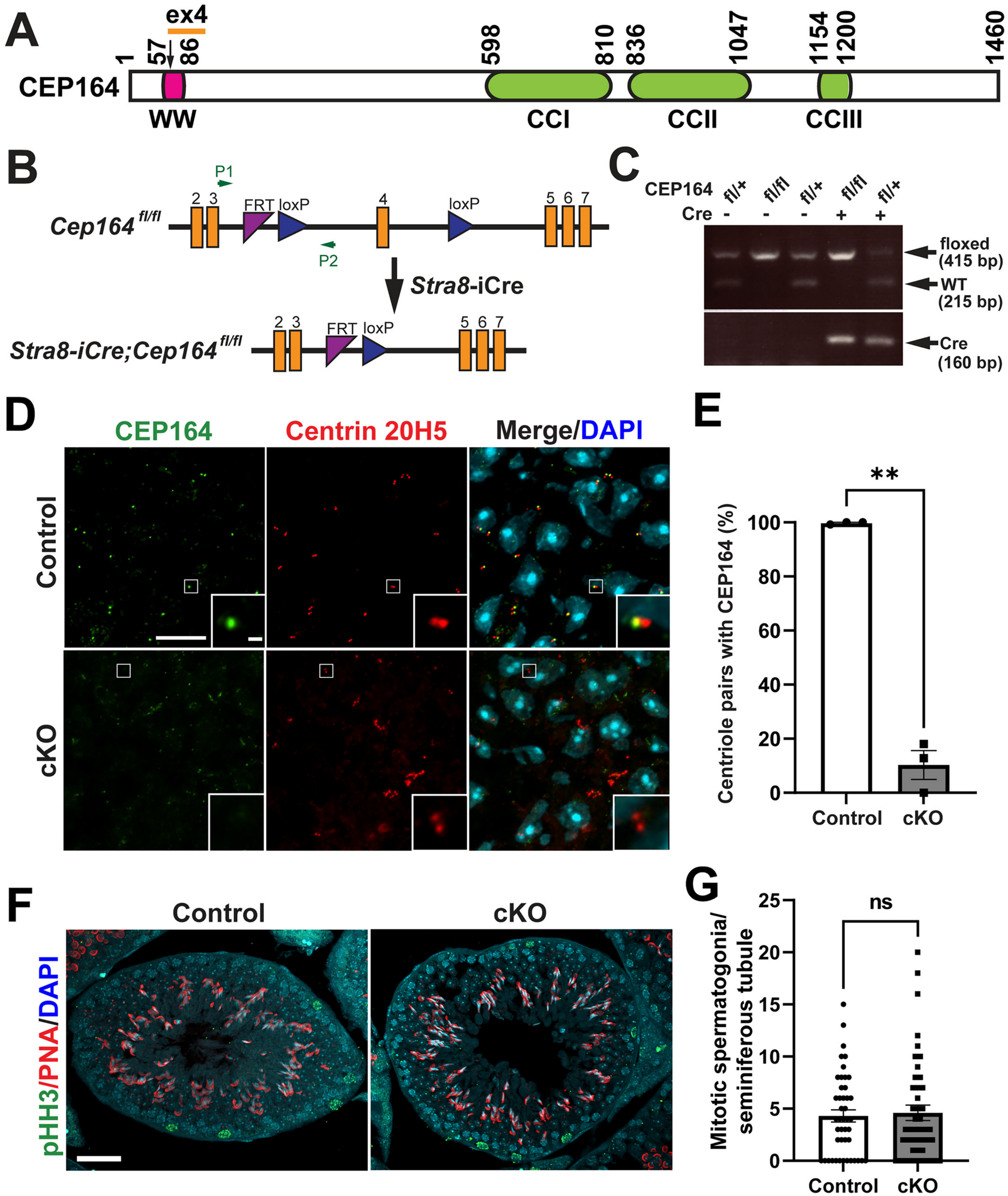
A
ex4
1
57
86
598
810
836
1047
1154
1200
1460
CEP164
WW
CCI
CCII
CCIII
B
P1
2 3
FRT loxP
4
loxP
5 6 7
Cep164 fl/fl
P2
Stra8-iCre
Stra8-iCre;Cep164 fl/fl
C
CEP164 fl/+ fl/fl fl/+ fl/fl fl/+
Cre - - - + +
floxed (415 bp)
WT (215 bp)
Cre (160 bp)
D
CEP164
Centrin 20H5
Merge/DAPI
Control
cKO
E
**
Centriole pairs with CEP164 (%)
100
80
60
40
20
0
Control
cKO
F
Control
cKO
pHH3/PNA/DAPI
G
ns
Mitotic spermatogonia/ seminiferous tubule
25
20
15
10
5
0
Control
cKO

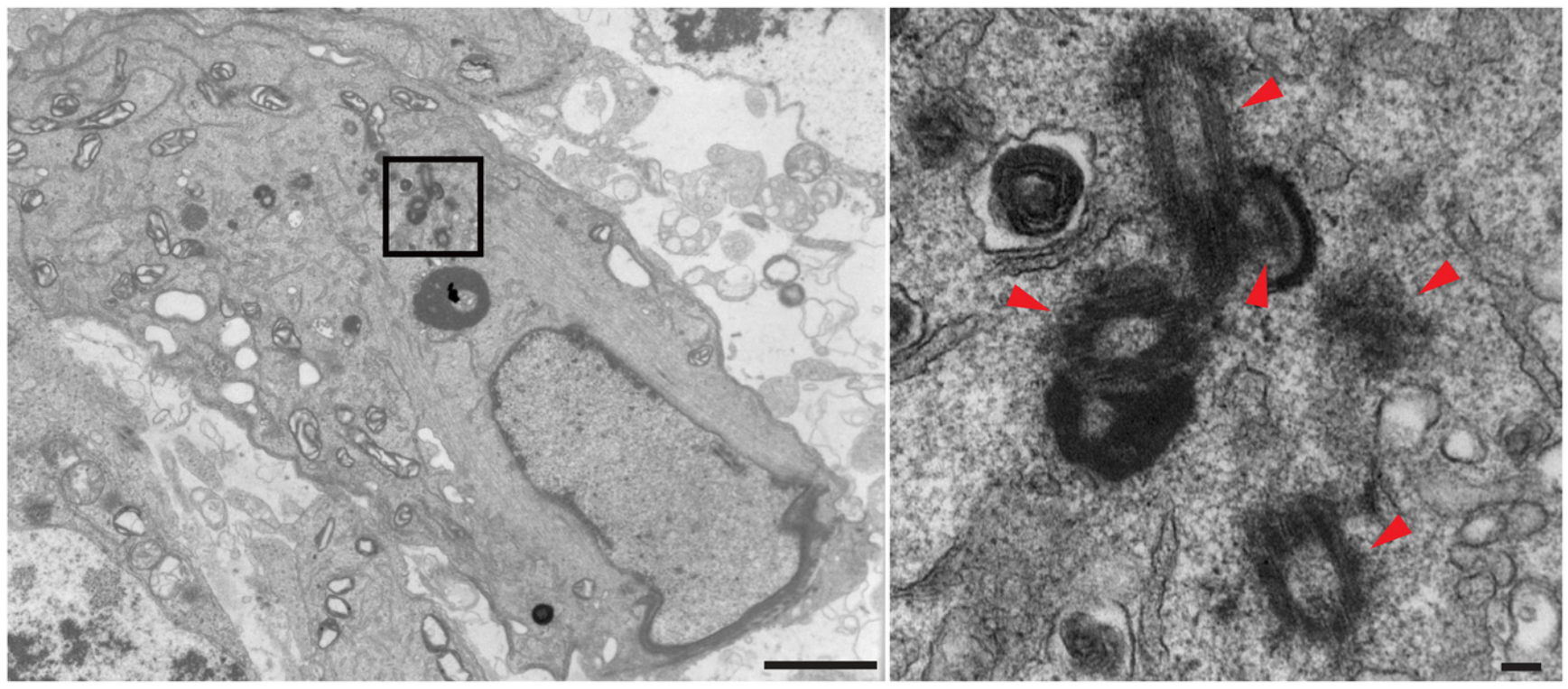

Figure EV2. TEM image of undocked supernumerary centrioles in a cKO spermatid.

TEM image of a P26 testis section showing a step 9 cKO spermatid containing five centrioles (red arrowheads). An enlarged view of the boxed region is shown on the right. Scale bars, 2 µm and 100 nm (enlarged images).

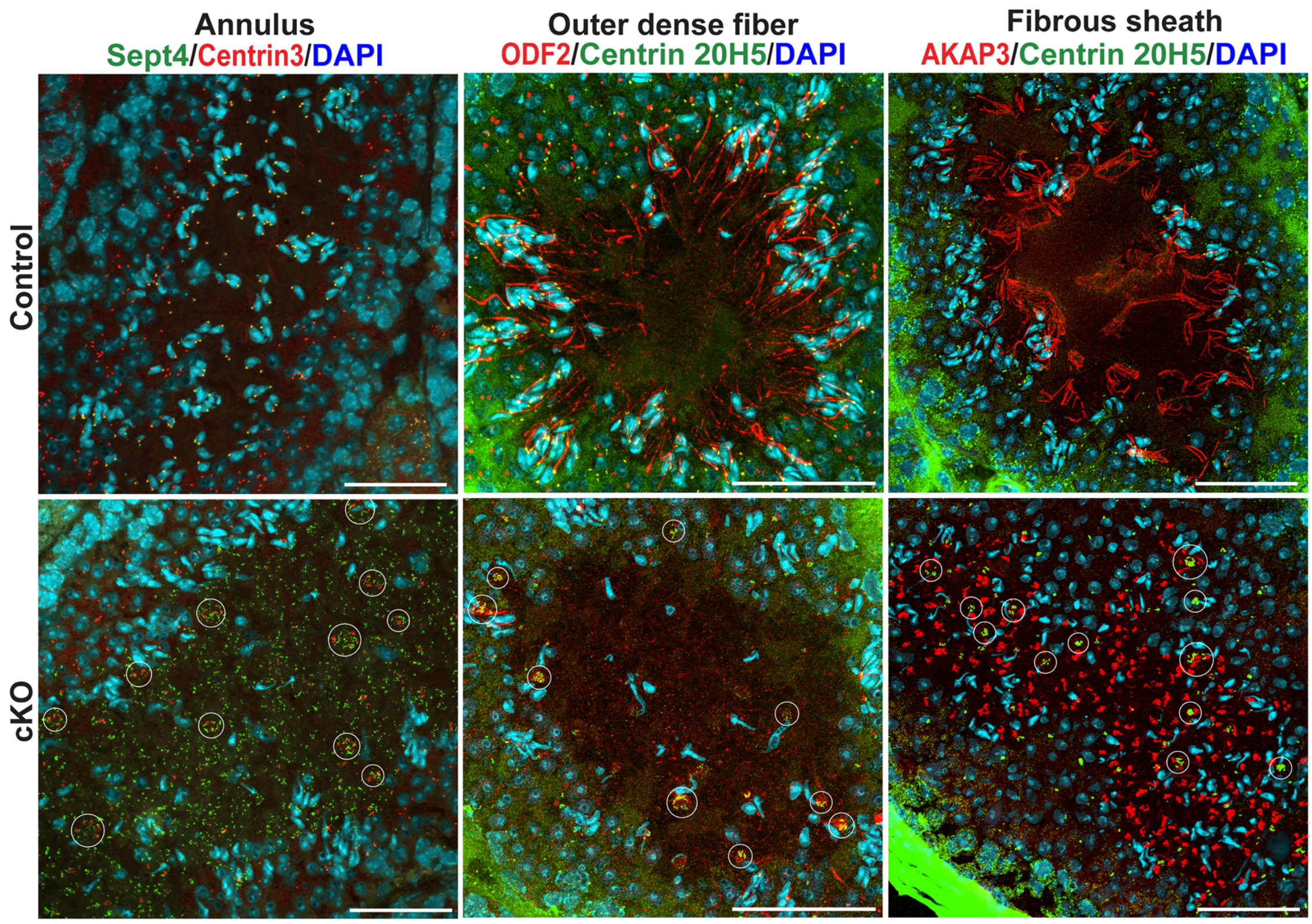

Figure EV3. IF analysis of sperm tail structural components in adult testes.

Adult testis frozen sections were immunostained using the following antibody combinations: Centrin 3 and Sept4 (annulus component), Centrin 20H5 and ODF2 (outer dense fiber component), and Centrin 20H5 and AKAP3 (fibrous sheath component) as indicated. Centriole clusters are outlined by circles. Scale bars, 50 µm.

 © The Author(s)

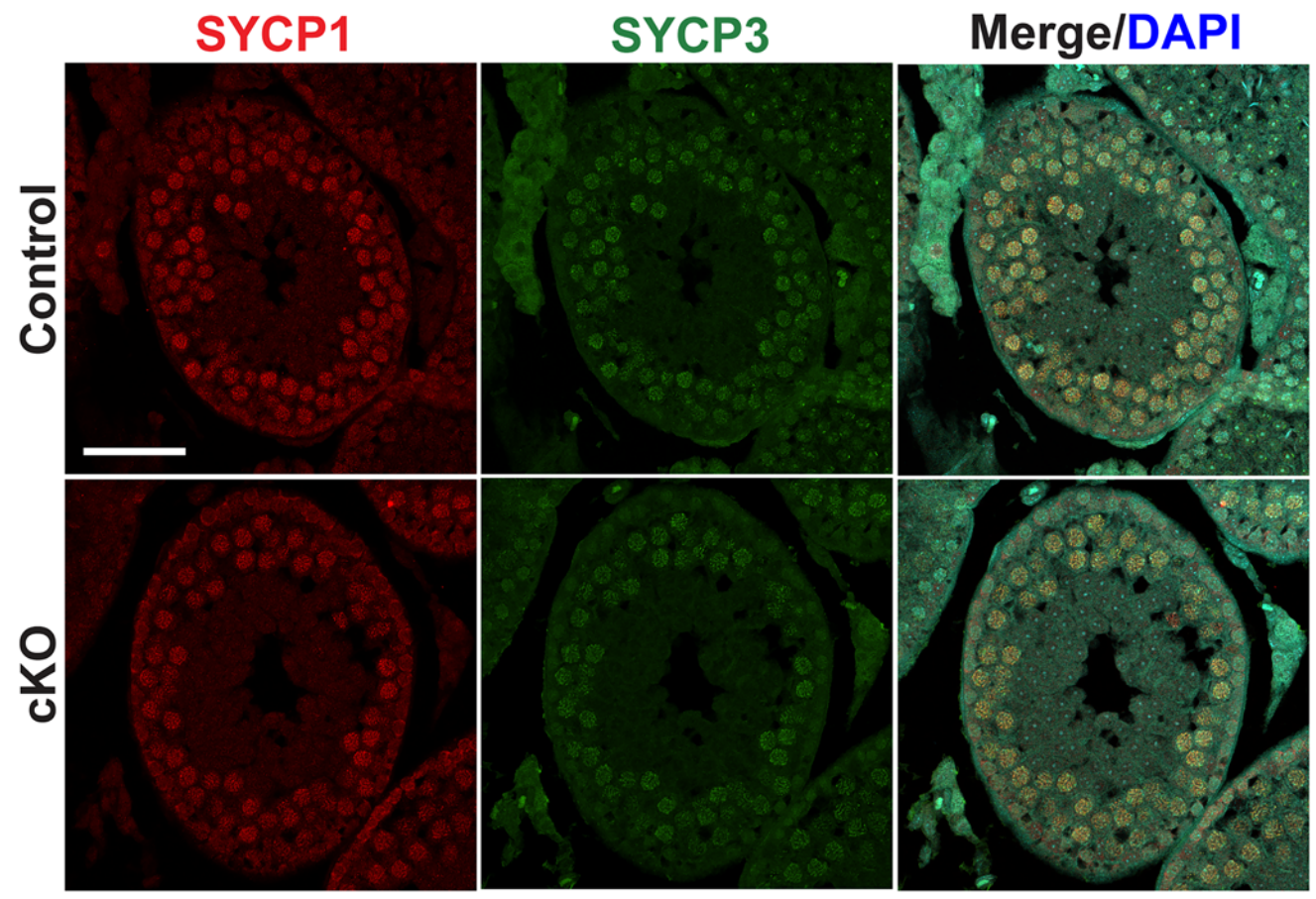

Figure EV4. Representative IF images of P25 testis sections immunostained for SYCP1 and SYCP3.

Testis paraffin sections from P25 littermates during the synchronized first wave of spermatogenesis were immunostained for SYCP1 and SYCP3. Scale bars, 50 µm.
