# Supplementary material for: Basal body docking failure triggers centriole clustering and elimination in mammalian spermatogenesis
Source: EMBO Rep. 2026 Jul 18;27(17):5039–61. doi: 10.1038/s44319-026-00872-8 (PMC13554106; doi:10.1038/s44319-026-00872-8)
Supplement: Supplementary file 2 [file 44319_2026_872_MOESM1_ESM.pdf]

EMBO reports

Basal body docking failure triggers centriole clustering and elimination in mammalian spermatogenesis

Jun Jie Chen, Xiangyu Gong, Michael Mak, Feng-Qian Li, and Ken-Ichi Takemaru

Corresponding author(s): Ken-Ichi Takemaru (ken-ichi.takemaru@stonybrook.edu)

**Review Timeline:**		
	Submission Date:	24th Oct 25
	Editorial Decision:	2nd Jan 26
	Revision Received:	14th Apr 26
	Editorial Decision:	19th May 26
	Revision Received:	22nd May 26
	Accepted:	3rd Jul 26

Editor: Kurt Weir

Transaction Report:

(Note: With the exception of the correction of typographical or spelling errors that could be a source of ambiguity, letters and reports are not edited. Depending on transfer agreements, referee reports obtained elsewhere may or may not be included in this compilation. Referee reports are anonymous unless the Referee chooses to sign their reports.)

1st Editorial Decision **2nd Jan 2026**

Dear Dr. Takemaru

Thank you for the submission of your research manuscript to our journal. We have now received the full set of referee reports that is copied below.

As you have seen, the referees acknowledge that the findings are interesting and that the conclusions are overall supported by the data presented but they also raise a number of concerns and have suggestions how to further strengthen the data, particularly more direct evidence of centriolar passage through intercellular bridges, relatively low number of replicates for some experiments, and an overstated title.

Given these constructive comments, we would like to invite you to revise your manuscript with the understanding that the referee concerns (as detailed above and in their reports) must be fully addressed and their suggestions taken on board. Please address all referee concerns in a complete point-by-point response. Acceptance of the manuscript will depend on a positive outcome of a second round of review. It is EMBO Reports policy to allow a single round of revision only and acceptance or rejection of the manuscript will therefore depend on the completeness of your responses included in the next, final version of the manuscript.

We realize that it is difficult to revise to a specific deadline. In the interest of protecting the conceptual advance provided by the work, we recommend a revision within 3 months (April 2nd). Please discuss the revision progress ahead of this time with the editor if you require more time to complete the revisions.

I am also happy to discuss the revision further via e-mail or a video call, if you wish.

You can either publish the study as a short report or as a full article. For short reports, the revised manuscript should not exceed 27,000 characters (including spaces but excluding materials & methods and references) and 5 main plus 5 expanded view figures. The results and discussion sections must further be combined, which will help to shorten the manuscript text by eliminating some redundancy that is inevitable when discussing the same experiments twice. For a normal article there are no length limitations, but it should have more than 5 main figures and the results and discussion sections must be separate. In both cases, the entire materials and methods must be included in the main manuscript file.

================
IMPORTANT NOTE:

We perform an initial quality control of all revised manuscripts before re-review. Your manuscript will FAIL this control and the handling will be delayed IN CASE the following APPLIES:

1) A data availability section providing access to data deposited in public databases is missing. If you have not deposited any data, please add a sentence to the data availability section that explains that.

2) Your manuscript contains statistics and error bars based on n=2. Please use scatter blots in these cases. No statistics should be calculated if n=2.

When submitting your revised manuscript, please carefully review the instructions that follow below. Failure to include requested items will delay the evaluation of your revision.

================

When submitting your revised manuscript, we will require:

1) a .docx formatted version of the manuscript text (including legends for main figures, EV figures and tables). Please make sure that the changes are highlighted to be clearly visible.

2) individual production quality figure files as .eps, .tif, .jpg (one file per figure).
Please download our Figure Preparation Guidelines (figure preparation pdf) from our Author Guidelines pages https://www.embopress.org/page/journal/14693178/authorguide for more info on how to prepare your figures.

3) a .docx formatted letter INCLUDING the reviewers' reports and your detailed point-by-point responses to their comments. As part of the EMBO Press transparent editorial process, the point-by-point response is part of the Review Process File (RPF), which will be published alongside your paper.

4) a complete author checklist, which you can download from our author guidelines

(<https://www.embopress.org/page/journal/14693178/authorguide>). Please insert information in the checklist that is also reflected in the manuscript. The completed author checklist will also be part of the RPF.

5) Please note that all corresponding authors are required to supply an ORCID ID for their name upon submission of a revised manuscript (<https://orcid.org/>). Please find instructions on how to link your ORCID ID to your account in our manuscript tracking system in our Author guidelines
(<https://www.embopress.org/page/journal/14693178/authorguide#authorshipguidelines>)

6) We replaced Supplementary Information with Expanded View (EV) Figures and Tables that are collapsible/expandable online. A maximum of 5 EV Figures can be typeset. EV Figures should be cited as 'Figure EV1, Figure EV2" etc... in the text and their respective legends should be included in the main text after the legends of regular figures.

- For the figures that you do NOT wish to display as Expanded View figures, they should be bundled together with their legends in a single PDF file called *Appendix*, which should start with a short Table of Content. Appendix figures should be referred to in the main text as: "Appendix Figure S1, Appendix Figure S2" etc. See detailed instructions regarding expanded view here:
<https://www.embopress.org/page/journal/14693178/authorguide#expandedview>

- Additional Tables/Datasets should be labeled and referred to as Table EV1, Dataset EV1, etc. Legends have to be provided in a separate tab in case of .xls files. Alternatively, the legend can be supplied as a separate text file (README) and zipped together with the Table/Dataset file.

7) Please include a dedicated "Data Availability" section at the end of the Methods (suggested wording: "The [structural coordinates | microarray | mass spectrometry] data from this publication have been deposited to the [name of the database] database [URL] and assigned the identifier [accession | permalink | hashtag]."). Should this not apply, this should still be stated as "This study includes no data deposited in external repositories."

8) At EMBO Press we ask authors to provide source data for the main manuscript figures. You will receive a separate email with instructions for providing source data with your revised manuscript, including how to upload and organize the files.

Additional information on source data and instruction on how to label the files are available
<https://www.embopress.org/page/journal/14693178/authorguide#sourcedata>

9) The journal requires a statement specifying whether or not authors have competing interests (defined as all potential or actual interests that could be perceived to influence the presentation or interpretation of an article). In case of competing interests, this must be specified in your disclosure statement. Further information: https://www.embopress.org/competing-interests

10) Figure legends and data quantification:
The following points must be specified in each figure legend:

- the name of the statistical test used to generate error bars and P values,
- the EXACT p-values,
- the number (n) of independent experiments (please specify technical or biological replicates) underlying each data point,
- the nature of the bars and error bars (s.d., s.e.m.)

- If the data are obtained from n {less than or equal to} 5, show the individual data points in addition to the SD or SEM.
- If the data are obtained from n {less than or equal to} 2, use scatter blots showing the individual data points.

Discussion of statistical methodology can be reported in the materials and methods section, but figure legends should contain a basic description of n, P and the test applied.

See also the guidelines for figure legend preparation:
https://www.embopress.org/page/journal/14693178/authorguide#figureformat

- Please also include scale bars in all microscopy images and define their size in the legend, not the image.

11) Our journal encourages inclusion of *data citations in the reference list* to directly cite datasets that were re-used and obtained from public databases. Data citations in the article text are distinct from normal bibliographical citations and should directly link to the database records from which the data can be accessed. In the main text, data citations are formatted as follows: "Data ref: Smith et al, 2001" or "Data ref: NCBI Sequence Read Archive PRJNA342805, 2017". In the Reference list, data citations must be labeled with "[DATASET]". A data reference must provide the database name, accession number/identifiers and a resolvable link to the landing page from which the data can be accessed at the end of the reference. Further instructions are available at <https://www.embopress.org/page/journal/14693178/authorguide#referencesformat>.

12) All Materials and Methods need to be described in the main text using our 'Structured Methods' format. According to this

format, the Methods section includes a Reagents and Tools Table (listing key reagents, experimental models, software and relevant equipment and including their sources and relevant identifiers) followed by a Methods and Protocols section describing the methods, ideally using a step-by-step protocol format. The aim is to facilitate adoption of the methodologies across labs. Please download and fill our Reagents and Tools Table template (.docx), which you can find in our author guidelines: https://www.embopress.org/page/journal/14693178/authorguide#structuredmethods.
When submitting your revised manuscript, please do not include the Reagents and Tools Table in the Methods section of the manuscript but upload it as a separate file choosing the file type "Reagent Table".
An example of a Method paper with Structured Methods can be found here:
https://www.embopress.org/doi/10.15252/msb.20178071.

13) As part of the EMBO publication's Transparent Editorial Process, EMBO Reports publishes online a Review Process File to accompany accepted manuscripts. This File will be published in conjunction with your paper and will include the referee reports, your point-by-point response and all pertinent correspondence relating to the manuscript.

You are able to opt out of this by letting the editorial office know (emboreports@embo.org). If you do opt out, the Review Process File link will point to the following statement: "No Review Process File is available with this article, as the authors have chosen not to make the review process public in this case."

We would also welcome the submission of cover suggestions, or motifs to be used by our Graphics Illustrator in designing a cover.

I look forward to seeing a revised form of your manuscript when it is ready and please let me know if you have questions or comments regarding the revision.

Yours sincerely,

Kurt Weir
Editor
EMBO Reports

Referee #1:

The manuscript by Chen et al. examines the role of the distal appendage protein CEP164 and primary cilia in mammalian spermatogenesis using a male germ cell-specific knockout mouse. The study demonstrates a clear cell-autonomous requirement for CEP164 in male germ cells. CEP164-cKO males are infertile despite normal testis size, and the loss of CEP164 eliminates both zygotene primary cilia in spermatocytes and sperm flagella. The authors report a sharp reduction in mature sperm recovered from the cauda epididymis.

Male infertility linked to CEP164 deficiency was previously reported by this group. Here, they separate CEP164's function in the sperm cells from its role in multiciliogenesis in the reproductive ducts, providing important insights for both cell biology and reproduction.

Although zygotene cilia are absent in the knockout, meiotic chromosome pairing, synapsis, and DSB repair appear unaffected. This differs from zebrafish, where loss of zygotene cilia disrupts bouquet formation, DSB repair, and fertility. In mice, infertility arises from defects in later stages, basal body docking and flagellogenesis, rather than meiosis.
The frequent formation of supernumerary centrioles is a striking finding, especially the report of up to 23 centrioles in a single cell.

Major Concerns

1- The authors show that only ~18.5% of control zygotene spermatocytes have detectable primary cilia, and in cKO this drops to 0%. However, Stra8-iCre is not 100% efficient, and CEP164 protein may persist in some cells (Fig EV1D shows residual staining). More importantly, the small subpopulation that normally ciliated might be the only ones that truly require cilia for bouquet function or DSB repair. The authors do not prove that the 18.5% that normally have cilia still lack them in the cKO.

2- Speaking frankly, I have no problem with conclusions but a minimal sample size is an issue. For example, the experiments central to the conclusion of organismal divergence, that meiotic progression is normal in mice, relied on minimal sampling. The quantification of Rad51 and gamma H2AX foci was based on a minimal sample size of n=3 mice.

3- While the live-imaging data (Movies EV1-2) are striking, strongly implies migration and clustering, the mechanistic basis is

underdeveloped and definitive evidence requires capturing the moment of centriole passage through the intercellular bridge itself. The authors propose movement through intercellular bridges, but they do not show direct evidence.

4- Although the authors confirm the absence of apoptosis markers, the conclusion that these acentriolar cells are removed by Sertoli-mediated phagocytosis remains observational (based on the presence of remnants in the lumen). The authors claim aggregates are "ultimately discarded into residual bodies," but they only show aggregates in round/elongating spermatids and tail-component-positive debris in the lumen. They do not track the fate of individual aggregates over time.

5- CEP164 has reported (though controversial) roles in DNA-damage response. The authors dismiss this quickly, but Stra8-iCre deletes early (type A spermatogonia), so prolonged absence of CEP164 could cause subtle centrosomal or cell-cycle defects that indirectly affect centriole docking rather than docking failing solely because of lost distal-appendage function.

Minor Concerns

5- The title is misleading/overstated: "Loss of cilia drives centriole clustering..." - actually, loss of basal-body docking (not loss of the cilium/axoneme per se) drives clustering. In zygotene cells the cilium is lost but centrioles do not cluster; clustering only occurs later in spermatids when docking to the plasma membrane fails.

6- The Methods section (Page 18) states: "Cep164fl/fl mice were used as controls throughout the study, except for Fig 6G, in which P25 Stra8-iCre; Cep164fl/+ and P26 Cep164fl/+ littermates were used as controls.". While the use of these heterozygous controls (Cep164fl/+) is justifiable (as they are littermates and are expected to be wild-type for the phenotype), the phrasing is slightly confusing, as it lists two different genotypes (P25 Stra8-iCre; Cep164fl/+ and Cep164fl/+) and two different ages (P25 and P26) as the control group for a single experiment (Fig 6G). The authors should clarify whether the data points for Fig 6G were pooled from these two distinct control groups.

7- Please discuss why only ~20% of zygotene cells ciliated in mice vs nearly 100% in zebrafish

8- Missing quantification of knockout efficiency in spermatocytes vs spermatids. Fig EV1D shows residual CEP164 staining, but no quantification by stage.

9- No sperm morphology analysis from the few sperm that occasionally appear. Very rare sperm are sometimes seen in the epididymis of severe flagellar mutants. It would be useful to know whether any sperm that escape the phenotype have normal centriole number and docking.

10- Figure legends and text inconsistently refer to "cKO" vs "Cep164 cKO". Minor but should be standardized.

11- No mention of potential female phenotype. Stra8-iCre is also active in females; even a brief statement that female fertility was or was not tested would be helpful.

12- The manchette elongation phenotype is shown but not discussed in depth This is a phenotype in many ciliopathy/centrosome mutants, yet the authors do not cite or discuss possible mechanisms.

Addressing the suggestions above would further solidify these significant contributions to the literature.

Referee #2:

Overview:
Although aspects of spermatogenesis are conserved across animal species, some of the detailed mechanisms are species-specific, even when they involve conserved proteins. An example of this is the ciliary distal appendage protein Cep164, which is dispensable for sperm development in Drosophila and has been reported to be involved in meiotic chromosome pairing and double-strand DNA break repair in zebrafish (although this is controversial). Here, the authors examined the role of Cep164 in mice. Although mouse male germ cells lacking Cep164 undergo meiosis and double-stranded DNA break repair properly, they exhibit later defects in basal body docking and flagellogenesis that is accompanied by formation of supernumerary, highly mobile centrioles that cluster together and are eliminated along with aberrantly structured sperm.
Overall, the experiments are nicely done, the data are strong, and the conclusions appear well justified. Moreover, the discussion makes interesting points and raises questions that are relevant to the field. However, there are a few aspects of the text and figures that need addressing before publication. These are listed below.

Text:
p. 5, five lines from bottom: Change "significantly" to "significant".
p. 8: The authors refer to data in Fig. 2 showing "abnormally elongated sperm heads" and persistence and elongation of the

manchette. It would be helpful to quantify the degree of elongation of the sperm heads and the manchette and also pinpoint how long the manchette persists beyond the stages where it is usually disassembled.
p. 10: Is there any significance to there being "as many as 23 centrioles per cell...in cKO testes"? How many spermatids are generally syncytially connected with each other, and does this have any relationship to the numbers of centrioles observed.

Figures:
Figure 2C: For the cKO, it would help to indicate where the high-mag images at the bottom come from if these are magnified from the images shown in the middle panels.
Figure 3D: Unlike the other graphs, the samples examine (n = 4) do not appear to be plotted on the graph. Is this because they all exhibited 100% syapsed autosomal chromosomes? This should be indicated in the figure or explained in the figure legend.
Figure 3E and G: Although the numbers of Rad51 and pH2AX puncta are similar between control and cKO samples, the images of cKO nuclei reveal larger Rad51 and pH2AX puncta than are found in the control. The authors should comment on whether this was typical of all samples of the indicated genotypes and whether the observed differences are likely meaningful.
Figure 4D: It would be helpful for the authors to include TEM images of basal bodies in control spermatids for comparison to those in the cKO spermatids. In addition, it appears that the magnified image in the lower right panel is inverted relative to the lower mag image on the left (white on gray rather than black on gray). This should be clarified in the legend.
Figure 5A: To the non-expert, it might not be clear that Sept4 is forming rings, as these are shown end-on rather than as cross-sectional views. This could be clarified in the legend.
Figure 5B legend: "20H5" does not need to be in brackets here (sole instance).
Figure 7A: The location of the centrioles relative to the plasma membrane is not clear, as the plasma membrane is barely (if at all) visible in the images. It would help to use a dye or stain of a different color to visualize the plasma membrane (FM dye, CellMask, etc.) to clarify the position of the centrioles relative to the membrane. Alternatively, it would help to draw dashed lines to mark the cell membranes.
In addition, in the images showing cKO spermatids, not only is the plasma membrane not evident, but it is unclear what the large subcellular structures are that are labeled with EGFP-Centrin1. Are these nuclei? This should be explained in the legend.
Movies EV1 and EV2: As with the still images, the location of the plasma membrane in these cells is not at all obvious in the movies. As described above, use of a plasma membrane dye, marker or stain would help make the observed phenotypes more obvious.

Referee #3:

The manuscript by Chen and colleagues examines the importance of CEP164 in spermatogenesis through examination of a tissue-specific knockout that causes infertility. In addition to quantifying the defects in flagella formation the authors found that centrioles moved between cells through intercellular bridges, often collecting in large clusters. This work is well executed and clearly explained.
The authors interpretation of the results was largely centered on the role of cilia in spermatogenesis. This is valuable and appropriate for publication in EMBO Reports. I suggest that there is an additional important take away from this paper. While centriole docking is essential for ciliogenesis, not all docked centrioles form cilia. This was shown in T-cells that dock centrioles when engaging target cells (Stinchcombe et al, 2006). More recently, cerebellar granule cell neurons were found to have docked centrioles and no cilia (Ott et al, 2024). In zygotene spermatocytes, 80% of the cells don't have cilia, yet the consequences of CEP164 loss are widespread. I think you have discovered another new, cilia-independent function for docked centrioles.
There is only one major concern that must be addressed to support the publication criteria for strong evidence for the conclusions drawn. The time resolution of the live cell imaging experiments is not sufficient to follow individual centrioles. The conclusion that the centriole amplification is not happening is supported by these experiments, but it is difficult to conclude anything about individual centriole movement. Shorter time intervals could bleach the sample more quickly, but you don't need to image as long to generate data to support your claims about centriole movement. If you could visualize the intracellular bridges at the same time, that would be excellent (I don't know if this is possible using transmitted light like DIC).

Minor
1- Figure EV1C - the first and third lane are labeled the same but look different. Please clarify if something is different or explain why they don't look the same.
2 - Please include the ROSA-EGFP-Centrin1 crosses in your Methods
3 - In your TEM - How many examples of centrioles did you find? Can you add any quantitation?
4 - Figure 2 -The top and center row look comparable. What area of the control is comparable to the bottom row?
Reviewed by Carolyn Ott

Ott CM, Constable S, Nguyen TM, White K, Lee W-CA, Lippincott-Schwartz J & Mukhopadhyay S (2024) Permanent deconstruction of intracellular primary cilia in differentiating granule cell neurons. J Cell Biol 223: e202404038
Stinchcombe JC, Majorovits E, Bossi G, Fuller S & Griffiths GM (2006) Centrosome polarization delivers secretory granules to the immunological synapse. Nature 443: 462-465

Referee #4:

Chen et al. report that CEP164 is required for centriole docking in mouse spermatids to drive axoneme (and thereby flagellum) formation and ultimately male fertility. They generated a pre-meiotic male germ cell-specific knockout of Cep164 (cKO) and found that it is required for axoneme biogenesis in zygotene spermatocytes and round spermatids. Axonemes/flagella are essential for round spermatid development, however, there are no overt consequences caused by their absence in cKO zygotene spermatocytes. Moreover, in the absence of centriole docking in spermatids, the authors find that centrioles are highly motile, and migrate between neighbouring cells to form clusters. Finally they uncover a novel mechanism that eliminates acentriolar spermatids during spermiogenesis. This manuscript provides significant findings and overall, is well-written and experiments have been thoughtfully considered and well-conducted. Given the importance of CEP164 across cell types and species, these findings will be of interest for cell biologist broadly, but particularly so for sperm cell biologists.

Minor comments:
1. Change the manuscript title to more accurately reflecting the main finding - the loss of cilia does not drive this mechanism per se, but the absence of centriole docking to plasma/nuclear membranes.
2. Fig 1D: please provide higher resolution images.
3. Across all testis section/isolated spermatid images, please state the seminiferous tubule Stage/spermatid step when making comparisons between control and cKO populations. Comparisons also need to be Stage-matched (e.g. Fig 2C is not stage matched, evident by spermatocytes in control (Stage XI) and round spermatids in cKO top panel (between Stages VI-VIII).
4. Provide details on the ROSA-EGFP-Centrin1 x Cep164fl/fl cross in the methods section.
5. Fig 4D: it is difficult to see the magnified micrograph on the bottom right panel.
6. How many cells were examined for Fig 4E (mother:daughter centriole ratio)?
7. Fig 5A: DAPI is missing in the bottom cKO merged panel
8. ODF2/outer dense fibres are not only in the midpiece, but also in the principal piece. Please update the sentence to reflect this.
9. The live cell imaging is very impressive given the constrains of working with this cell type. I think the hypothesis that centrioles move through ICBs is highly plausible but that it has not been directly shown here, I suggest updating 7B to clearly state this is a proposed mechanism/hypothesis.

We are grateful to the Editor and all four Referees for their rigorous and insightful critiques. Their comments provided an excellent opportunity to further refine and strengthen the manuscript. We provide detailed, point-by-point responses below.

Referee 1

The manuscript by Chen et al. examines the role of the distal appendage protein CEP164 and primary cilia in mammalian spermatogenesis using a male germ cell-specific knockout mouse. The study demonstrates a clear cell-autonomous requirement for CEP164 in male germ cells. CEP164-cKO males are infertile despite normal testis size, and the loss of CEP164 eliminates both zygotene primary cilia in spermatocytes and sperm flagella. The authors report a sharp reduction in mature sperm recovered from the cauda epididymis.

Male infertility linked to CEP164 deficiency was previously reported by this group. Here, they separate CEP164's function in the sperm cells from its role in multiciliogenesis in the reproductive ducts, providing important insights for both cell biology and reproduction.

Although zygotene cilia are absent in the knockout, meiotic chromosome pairing, synapsis, and DSB repair appear unaffected. This differs from zebrafish, where loss of zygotene cilia disrupts bouquet formation, DSB repair, and fertility. In mice, infertility arises from defects in later stages, basal body docking and flagellogenesis, rather than meiosis.
The frequent formation of supernumerary centrioles is a striking finding, especially the report of up to 23 centrioles in a single cell.

Major Concerns

1- The authors show that only ~18.5% of control zygotene spermatocytes have detectable primary cilia, and in cKO this drops to 0%. However, Stra8-iCre is not 100% efficient, and CEP164 protein may persist in some cells (Fig EV1D shows residual staining). More importantly, the small subpopulation that normally ciliated might be the only ones that truly require cilia for bouquet function or DSB repair. The authors do not prove that the 18.5% that normally have cilia still lack them in the cKO.
We thank the Referee for this thoughtful and important comment. We respectfully note that it is technically challenging to determine which specific zygotene spermatocytes are destined to develop primary cilia, as there are currently no established markers that distinguish ciliated from non-ciliated zygotene cells. To further support our conclusions in response to this concern, we expanded our analysis by increasing the number of biological replicates (controls: n = 4; cKO: increased from n = 3 to n = 4) and by increasing the number of zygotene spermatocytes quantified per animal (from 100 to 150 zygotene spermatocytes per mouse). With this expanded dataset, we identified only 6 ciliated cells out of 600 zygotene spermatocytes in cKO testes (1.0%), compared to 115 ciliated cells out of 600 in controls (19.2%), representing an approximately 95% reduction. Our quantitative data suggest that primary cilia are eliminated in the majority of zygotene spermatocytes in the cKO testes. Furthermore, we have added the following sentence to the Discussion section (page 17): “Although we can not formally exclude the possibility that a small number of residual zygotene cilia may be sufficient to support these processes, potentially through syncytial communication between germ cells connected by intercellular bridges, our findings nevertheless suggest that zygotene cilia are dispensable for proper progression of male meiosis in mice”.

2- Speaking frankly, I have no problem with conclusions but a minimal sample size is an issue.

For example, the experiments central to the conclusion of organismal divergence, that meiotic progression is normal in mice, relied on minimal sampling. The quantification of Rad51 and gamma H2AX foci was based on a minimal sample size of n=3 mice.
We agree with the Referee. In response to this concern, we have increased the number of biological replicates from n = 3 to n = 4 and expanded the number of cells quantified from 10 to 15 per animal for Fig. 3F,H. In addition, for Fig. 6G, we increased the number of biological replicates from n = 2 to n = 3, which now meets statistical significance.

3- While the live-imaging data (Movies EV1-2) are striking, strongly implies migration and clustering, the mechanistic basis is underdeveloped and definitive evidence requires capturing the moment of centriole passage through the intercellular bridge itself. The authors propose movement through intercellular bridges, but they do not show direct evidence.
We thank the Referee for the positive evaluation of our live-cell imaging data. We agree that centriole movement through intercellular bridges remains a proposed mechanism that has not been directly demonstrated in this study. Accordingly, we have tempered our interpretation and revised the legend to Fig. 7B to clearly indicate that this represents a hypothetical model. We have also clarified this point in the Discussion section (page 20): "However, due to technical limitations of our live-cell imaging approach, plasma membranes and intercellular bridges could not be directly visualized, and therefore it remains unclear whether centrioles physically transit through intercellular bridges".

4- Although the authors confirm the absence of apoptosis markers, the conclusion that these acentriolar cells are removed by Sertoli-mediated phagocytosis remains observational (based on the presence of remnants in the lumen). The authors claim aggregates are "ultimately discarded into residual bodies," but they only show aggregates in round/elongating spermatids and tail-component-positive debris in the lumen. They do not track the fate of individual aggregates over time.
We agree with the Referee and have toned down the statement and now present Sertoli cell-mediated phagocytosis as a possible mechanism. We have also added germ cell exfoliation as an addition alternative. The revised sentence now reads (page 15): "As no morphological signs of apoptosis were observed, and neither cleaved caspase-3 immunostaining nor TUNEL assays detected apoptotic cells, we speculate that abnormal spermatids in the cKO testis may instead may be eliminated through alternative mechanisms, such as Sertoli cell-mediated phagocytosis or exfoliation into the seminiferous tubule lumen (Franca *et al*, 2016; O'Donnell *et al*, 2022; San Agustin *et al.*, 2015)".
Regarding the centriole aggregates, we have examined the distribution of centriole aggregates across different stages of spermatogenesis and have added new images in new Fig. 5A to clarify this point. As spermiogenesis progresses, many centriole aggregates are observed at the distal tip of elongated manchette structures towards the seminiferous tubule lumen. These observations are consistent with the idea that the aggregates may be discarded via residual bodies. To avoid overinterpretation, we have also softened the wording in the Abstract (page 2) and Introduction (page 6).

5- CEP164 has reported (though controversial) roles in DNA-damage response. The authors dismiss this quickly, but Stra8-iCre deletes early (type A spermatogonia), so prolonged absence of CEP164 could cause subtle centrosomal or cell-cycle defects that indirectly affect centriole docking rather than docking failing solely because of lost distal-appendage function.
We thank the reviewer for this insightful comment. To address possible changes in centrosome function during mitosis and in the cell cycle, we assessed spermatogonial division by staining for phospho-histone H3 (Ser10), a mitotic marker. Representative images are shown in Fig. EV1F,

with quantification in Fig. EV1G. The mitotic index of spermatogonia was comparable between control and cKO testes. While we can not fully exclude subtle effects, these findings suggest that overall cell-cycle progression does not appear to be altered by loss of CEP164 in spermatogonial stem cells. These results have now been incorporated into the Results section (page 7).

Minor Concerns

5- The title is misleading/overstated: "Loss of cilia drives centriole clustering..." - actually, loss of basal-body docking (not loss of the cilium/axoneme per se) drives clustering. In zygotene cells the cilium is lost but centrioles do not cluster; clustering only occurs later in spermatids when docking to the plasma membrane fails.
We thank the Referee for the suggestion. We have revised the title to “Basal body docking failure triggers centriole clustering and elimination during mammalian spermatogenesis”.

6- The Methods section (Page 18) states: "Cep164fl/fl mice were used as controls throughout the study, except for Fig 6G, in which P25 Stra8-iCre; Cep164fl/+ and P26 Cep164fl/+ littermates were used as controls.". While the use of these heterozygous controls (Cep164fl/+) is justifiable (as they are littermates and are expected to be wild-type for the phenotype), the phrasing is slightly confusing, as it lists two different genotypes (P25 Stra8-iCre; Cep164fl/+ and Cep164fl/+) and two different ages (P25 and P26) as the control group for a single experiment (Fig 6G). The authors should clarify whether the data points for Fig 6G were pooled from these two distinct control groups.
We apologize for the confusion. To minimize experimental variability, for Fig. 6G, we used heterozygous *Cep164*$^{fl/+}$ mice with or without *Stra8*-iCre, as obtaining age-matched *Cep164*$^{fl/fl}$ littermate controls was also challenging. In addition, to improve statistical robustness, we have analyzed an additional P26 littermate pair (one cKO and one *Stra8-iCre;Cep164*$^{fl/+}$ control). Data from these animals were pooled and plotted together in Fig. 6G. Importantly, heterozygous mice, regardless of the presence of *Stra8*-iCre, are phenotypically normal with respect to testis morphology and fertility. The Methods section has been revised to clarify these points (page 23).

7- Please discuss why only ~20% of zygotene cells ciliated in mice vs nearly 100% in zebrafish
We now have provided possible explanations in the Discussion section (pages 17-18).

8- Missing quantification of knockout efficiency in spermatocytes vs spermatids. Fig EV1D shows residual CEP164 staining, but no quantification by stage.
The CEP164 antibody used in this study works reliably only in frozen sections. Under these conditions, CEP164 staining is robust in spermatids but not in spermatocytes. For this reason, we quantified CEP164 knockout efficiency in round spermatids at P20-23 (Fig. EV1E). In addition, we have replaced the images in Fig. EV1D with lower-magnification views to include more cells. Quantification showed that CEP164 was absent from the mother centriole in 89% of cKO round spermatids.

9- No sperm morphology analysis from the few sperm that occasionally appear. Very rare sperm are sometimes seen in the epididymis of severe flagellar mutants. It would be useful to know whether any sperm that escape the phenotype have normal centriole number and docking.
To address this point, we performed IF staining of caput epididymal sections using antibodies against acetylated α-tubulin and γ-tubulin, together with PNA and DAPI counterstaining. The new data are presented in Fig. 1E. As expected, control epididymides contained abundant sperm, whereas the majority of cKO epididymides were largely devoid of sperm. Only rare

sperm and occasional abnormal round cells were observed. Notably, some of these round cells contained centriole clusters. Although the γ-tubulin antibody detected centrioles in epididymal epithelial cells and centriole clusters in abnormal round cells, it did not reliably label centrioles in mature sperm under these conditions. In addition, we have included new H&E images demonstrating the presence of abnormal round cells within the cKO caput epididymal tubules (Fig. 1D). We also examined mature sperm isolated from the cauda epididymis and found that most exhibited normal morphology with elongated flagella, suggesting that these likely represent escapers from Cre-mediated recombination (data not shown).

10- Figure legends and text inconsistently refer to "cKO" vs "Cep164 cKO". Minor but should be standardized.
We have carefully checked the manuscript and standardized the terminology throughout to cKO.

11- No mention of potential female phenotype. Stra8-iCre is also active in females; even a brief statement that female fertility was or was not tested would be helpful.
Stra8-iCre has been reported to show no functional Cre activity in female germ cells for reasons that remain unclear (PMID: 18850594). Therefore, assessment of a potential female germ cell phenotype was not feasible using this model. We have now clarified in the Discussion section that female fertility was not examined in this study (page 21).

12- The manchette elongation phenotype is shown but not discussed in depth This is a phenotype in many ciliopathy/centrosome mutants, yet the authors do not cite or discuss possible mechanisms.
In response to this point and a similar concern raised by Referee 2, we have now quantified both manchette length and spermatid head length. Representative IF images are shown in Fig. 2D, with the corresponding quantification presented in Fig. 2E. Our analysis demonstrates that both manchette length and spermatid head length are significantly increased in cKO spermatids compared with controls. In addition, we have investigated manchette dynamics across different spermatogenic stages (new Fig. 5A). Furthermore, we have expanded the Discussion section to include possible mechanisms underlying manchette elongation, with references (page 19).

Addressing the suggestions above would further solidify these significant contributions to the literature.

Referee 2

Overview:
Although aspects of spermatogenesis are conserved across animal species, some of the detailed mechanisms are species-specific, even when they involve conserved proteins. An example of this is the ciliary distal appendage protein Cep164, which is dispensable for sperm development in Drosophila and has been reported to be involved in meiotic chromosome pairing and double-strand DNA break repair in zebrafish (although this is controversial). Here, the authors examined the role of Cep164 in mice. Although mouse male germ cells lacking Cep164 undergo meiosis and double-stranded DNA break repair properly, they exhibit later defects in basal body docking and flagellogenesis that is accompanied by formation of supernumerary, highly mobile centrioles that cluster together and are eliminated along with aberrantly structured sperm.
Overall, the experiments are nicely done, the data are strong, and the conclusions appear well justified. Moreover, the discussion makes interesting points and raises questions that are

relevant to the field. However, there are a few aspects of the text and figures that need addressing before publication. These are listed below.

Text:
p. 5, five lines from bottom: Change "significantly" to "significant".
This has been corrected.

p. 8: The authors refer to data in Fig. 2 showing "abnormally elongated sperm heads" and persistence and elongation of the manchette. It would be helpful to quantify the degree of elongation of the sperm heads and the manchette and also pinpoint how long the manchette persists beyond the stages where it is usually disassembled.
We thank the Referee for this insightful point. In response, we have now quantified both manchette length and spermatid head length at stage XII. Representative IF images are shown in Fig. 2D, with the corresponding quantification presented in Fig. 2E. In mice, the manchette normally forms at step 8 and is disassembled around steps 13–14 (PMID: 26792866). Our analysis showed that both manchette length and sperm head length are significantly increased in cKO spermatids compared with controls. In addition, we have investigated manchette dynamics across different spermatogenic stages (new Fig. 5A). To our surprise, the overall timing of manchette assembly and disassembly did not appear substantially altered. We have revised the Results section (pages 9, 12, and 13) and Discussion section (page 19) accordingly to incorporate these new quantitative and qualitative findings.

p. 10: Is there any significance to there being "as many as 23 centrioles per cell...in cKO testes"? How many spermatids are generally syncytially connected with each other, and does this have any relationship to the numbers of centrioles observed.
Thank you for pointing out this important issue. The value reported here (up to 23 centrioles) represents the highest number we could confidently identify within a single cKO spermatid. In *Drosophila*, germ cell cyst organization is well defined: female germ cells form 16-cell cysts, whereas male germ cells form 64-cell cysts connected by intercellular bridges (PMID: 21669984). In mammals, however, this organization is considerably more complex, variable, and less clearly defined. During mammalian spermatogenesis, very large syncytia containing theoretically thousands of germ cells may form due to incomplete cytokinesis. In practice, however, studies in rat testes have reported syncytia containing up to approximately 650 interconnected germ cells (PMID: 1759679), which could theoretically correspond to as many as ~1,300 centrioles. The centriole clusters we detected typically consisted of up to 23 centrioles or potentially slightly more, suggesting that centriole clustering likely occurs within a limited subset of interconnected germ cells rather than across an entire syncytium in the cKO testis.

Figures:
Figure 2C: For the cKO, it would help to indicate where the high-mag images at the bottom come from if these are magnified from the images shown in the middle panels.
The IF images in Fig 2C have been replaced with a new set of images to ensure consistent seminiferous tubule staging (as suggested by the Referee 4). In addition, we have added boxes to indicate the regions shown in the higher-magnification images.

Figure 3D: Unlike the other graphs, the samples examine (n = 4) do not appear to be plotted on the graph. Is this because they all exhibited 100% syapsed autosomal chromosomes? This should be indicated in the figure or explained in the figure legend.
We apologize for the confusion. Yes, this is because all examined samples (30 pachytene cells per genotype) exhibited 100% synapsed autosomes. Because the values were identical, the

data points overlap in the graph and were therefore difficult to distinguish. We have clarified this point in the figure legend.

Figure 3E and G: Although the numbers of Rad51 and pH2AX puncta are similar between control and cKO samples, the images of cKO nuclei reveal larger Rad51 and pH2AX puncta than are found in the control. The authors should comment on whether this was typical of all samples of the indicated genotypes and whether the observed differences are likely meaningful.
To address this concern, we have replaced the cKO pH2AX images with more representative examples. We note that variability in foci size is observed in both control and cKO samples and is not unique to the mutant condition. Across multiple samples, we did not observe a consistent or reproducible increase in foci size specific to the cKO genotype. Therefore, we believe that the apparent difference in the original images likely reflects normal variability rather than a biologically meaningful change. We have now clarified this point in the figure legend.

Figure 4D: It would be helpful for the authors to include TEM images of basal bodies in control spermatids for comparison to those in the cKO spermatids. In addition, it appears that the magnified image in the lower right panel is inverted relative to the lower mag image on the left (white on gray rather than black on gray). This should be clarified in the legend.
We have now performed TEM analysis of control testis samples and included representative images of basal bodies from control spermatids in Fig. 4D for comparison with the cKO samples. Regarding the apparent inversion in contrast in the magnified image, this occurred during file conversion from Adobe Illustrator to PDF. This has now been corrected in the revised figure.

Figure 5A: To the non-expert, it might not be clear that Sept4 is forming rings, as these are shown end-on rather than as cross-sectional views. This could be clarified in the legend.
To clarify this point, we have edited the figure legend.

Figure 5B legend: "20H5" does not need to be in brackets here (sole instance).
This has been corrected.

Figure 7A: The location of the centrioles relative to the plasma membrane is not clear, as the plasma membrane is barely (if at all) visible in the images. It would help to use a dye or stain of a different color to visualize the plasma membrane (FM dye, CellMask, etc.) to clarify the position of the centrioles relative to the membrane. Alternatively, it would help to draw dashed lines to mark the cell membranes.
We performed live-cell imaging with DIC optics. However, the plasma membrane could not be reliably visualized in both control and cKO samples under our imaging conditions. We were also unable to further optimize the use of membrane dyes during the revision period because isolated spermatids are highly sensitive to experimental manipulation and exhibit poor viability in culture. In addition, we had only one cKO mouse expressing EGFP-Centrin1 available for revision experiments, as the yield of cKO mice is low. This may be due to previously unreported early embryonic expression of Stra8-iCre, which may cause embryonic lethality. Consistent with this possibility, we have occasionally observed cKO mice with polydactyly, suggesting embryonic Cre activity. Because of these technical limitations, we were unable to directly define the position of centrioles relative to the plasma membrane. Accordingly, we have toned down our interpretation and revised the legend to our model in Fig. 7B to clearly indicate that this represents a hypothetical model. We have also clarified this limitation in the Discussion section (page 20).

In addition, in the images showing cKO spermatids, not only is the plasma membrane not evident, but it is unclear what the large subcellular structures are that are labeled with EGFP-Centrin1. Are these nuclei? This should be explained in the legend.
We apologize for the confusion. The large round structures visible in these images are autofluorescent floating cells. We have clarified this point in the revised figure legend.

Movies EV1 and EV2: As with the still images, the location of the plasma membrane in these cells is not at all obvious in the movies. As described above, use of a plasma membrane dye, marker or stain would help make the observed phenotypes more obvious.
We thank the reviewer for this helpful suggestion. Please see our response to the comment on Figure 7A above, where we discuss this point and clarify the limitations of the current dataset.

Referee 3

The manuscript by Chen and colleagues examines the importance of CEP164 in spermatogenesis through examination of a tissue-specific knockout that causes infertility. In addition to quantifying the defects in flagella formation the authors found that centrioles moved between cells through intercellular bridges, often collecting in large clusters. This work is well executed and clearly explained.
The authors interpretation of the results was largely centered on the role of cilia in spermatogenesis. This is valuable and appropriate for publication in EMBO Reports. I suggest that there is an additional important take away from this paper. While centriole docking is essential for ciliogenesis, not all docked centrioles form cilia. This was shown in T-cells that dock centrioles when engaging target cells (Stinchcombe et al, 2006). More recently, cerebellar granule cell neurons were found to have docked centrioles and no cilia (Ott et al, 2024). In zygotene spermatocytes, 80% of the cells don't have cilia, yet the consequences of CEP164 loss are widespread. I think you have discovered another new, cilia-independent function for docked centrioles.
There is only one major concern that must be addressed to support the publication criteria for strong evidence for the conclusions drawn. The time resolution of the live cell imaging experiments is not sufficient to follow individual centrioles. The conclusion that the centriole amplification is not happening is supported by these experiments, but it is difficult to conclude anything about individual centriole movement. Shorter time intervals could bleach the sample more quickly, but you don't need to image as long to generate data to support your claims about centriole movement. If you could visualize the intracellular bridges at the same time, that would be excellent (I don't know if this is possible using transmitted light like DIC).
We thank Dr. Ott for her insightful and constructive comments and for highlighting important examples demonstrating that not all docked centrioles form cilia, such as in T cells (Stinchcombe et al., 2006) and cerebellar granule neurons (Ott et al., 2024). We agree that these observations support the emerging concept that docked centrioles may have cilia-independent functions. To better place our findings in this context, we have expanded the Discussion section to incorporate these examples and to further consider the possibility that CEP164 and centriole docking may also contribute to cilia-independent cellular processes (page 21).

We thank Dr. Ott for this important comment regarding the temporal resolution of our live-cell imaging experiments. In response, we performed additional live imaging with improved temporal resolution (16-second intervals for 15 minutes), instead of the 15-minute intervals used in the original time-lapse experiments. Consistent with our previous observations, centriole pairs in

control spermatids remained largely stationary, whereas centrioles in cKO spermatids exhibited pronounced mobility. These new data are now included as Movies EV3 and EV4, and the Results section has been revised accordingly (page 16).

We used DIC optics during live-cell imaging. However, intercellular bridges and plasma membranes could not be reliably visualized in either control or cKO spermatids under our imaging conditions. Accordingly, we have toned down our interpretation and revised the legend to the model in Fig. 7B to clearly indicate that this represents a hypothetical model rather than a directly demonstrated mechanism. We have also clarified this technical limitation in the Discussion section (page 20). Please also refer to our responses to related comments from the Referee 1 (Major Concern 3) and the Referee 2 (Figure 7A).

Minor

1- Figure EV1C - the first and third lane are labeled the same but look different. Please clarify if something is different or explain why they don't look the same.
The samples in lanes 1 and 3 have the same genotype (CEP164$^{fl/+}$) and therefore show two bands. For CEP164 genotyping, we used two primers (P1 and P2), as shown in Fig. EV1B. The single band in homozygous (fl/fl) samples is typically stronger, whereas the two bands in heterozygous (fl/+) samples appear weaker, but two distinct bands are visible. The two bands are more clearly visible in the digital image than in the printed version.

2 - Please include the ROSA-EGFP-Centrin1 crosses in your Methods
This has been described in the Methods section (page 23).

3 - In your TEM - How many examples of centrioles did you find? Can you add any quantitation?
Since TEM sections are thin (60-90 nm), we typically observed 3-4 centrioles per section. This likely reflects the fact that centriole clusters are distributed across different depths rather than lying within a single plane. As a result, precise quantification of centriole numbers based solely on TEM sections is technically challenging. However, to further support our observations, we have included additional TEM images showing a cKO spermatid containing 4 centrioles below (yellow arrowheads).

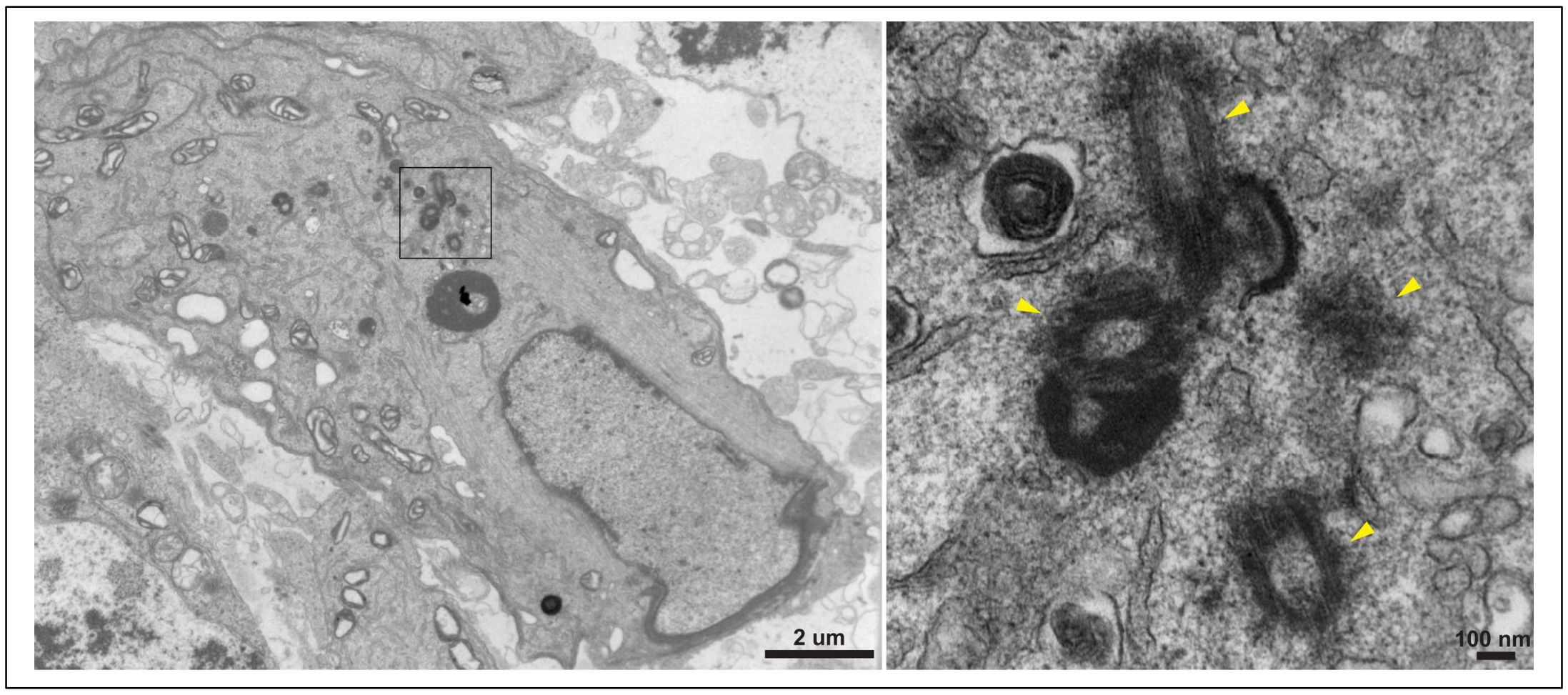

4 - Figure 2 -The top and center row look comparable. What area of the control is comparable to the bottom row?
We apologize for the confusion. The IF images in Fig 2C have now been replaced with a new set of images to ensure consistent seminiferous tubule staging (as suggested by the Referee 4).

In addition, we have added boxes to indicate the specific regions shown in the higher-magnification images.

Reviewed by Carolyn Ott

Ott CM, Constable S, Nguyen TM, White K, Lee W-CA, Lippincott-Schwartz J & Mukhopadhyay S (2024) Permanent deconstruction of intracellular primary cilia in differentiating granule cell neurons. J Cell Biol 223: e202404038
Stinchcombe JC, Majorovits E, Bossi G, Fuller S & Griffiths GM (2006) Centrosome polarization delivers secretory granules to the immunological synapse. Nature 443: 462-465

Referee 4

Chen et al. report that CEP164 is required for centriole docking in mouse spermatids to drive axoneme (and thereby flagellum) formation and ultimately male fertility. They generated a pre-meiotic male germ cell-specific knockout of Cep164 (cKO) and found that it is required for axoneme biogenesis in zygotene spermatocytes and round spermatids. Axonemes/flagella are essential for round spermatid development, however, there are no overt consequences caused by their absence in cKO zygotene spermatocytes. Moreover, in the absence of centriole docking in spermatids, the authors find that centrioles are highly motile, and migrate between neighbouring cells to form clusters. Finally they uncover a novel mechanism that eliminates acentriolar spermatids during spermiogenesis. This manuscript provides significant findings and overall, is well-written and experiments have been thoughtfully considered and well-conducted. Given the importance of CEP164 across cell types and species, these findings will be of interest for cell biologist broadly, but particularly so for sperm cell biologists.

Minor comments:
1. Change the manuscript title to more accurately reflecting the main finding - the loss of cilia does not drive this mechanism per se, but the absence of centriole docking to plasma/nuclear membranes.
We agree with the Referee. We have revised the title to better reflect the main finding of the study. The new title is: “Basal body docking failure triggers centriole clustering and elimination during mammalian spermatogenesis.”

2. Fig 1D: please provide higher resolution images.
We have now replaced the H&E images of the epididymis with higher-resolution images in Fig. 1D.

3. Across all testis section/isolated spermatid images, please state the seminiferous tubule Stage/spermatid step when making comparisons between control and cKO populations. Comparisons also need to be Stage-matched (e.g. Fig 2C is not stage matched, evident by spermatocytes in control (Stage XI) and round spermatids in cKO top panel (between Stages VI-VIII).
We thank the reviewer for this important comment. We have carefully re-examined the staging of seminiferous tubules and, where possible, have clarified the spermatogenesis stage or spermatid step for all relevant images. For comparisons where stage matching was critical, we have ensured that appropriately matched stages are shown and have clearly indicated this information in the figures and figure legends. In addition, we have provided new images where necessary to ensure stage-matched comparisons. We have also revised the text to improve clarity regarding staging.

4. Provide details on the ROSA-EGFP-Centrin1 x Cep164fl/fl cross in the methods section.
This has been described in the Methods section (page 23).

5. Fig 4D: it is difficult to see the magnified micrograph on the bottom right panel.
We apologize about the apparent inversion in contrast in the magnified image. This occurred during file conversion from Adobe Illustrator to PDF. This has now been corrected in the revised figure.

6. How many cells were examined for Fig 4E (mother:daughter centriole ratio)?
We examined 50 centriole pairs from control round spermatids and 58 centriole pairs from cKO round spermatids in adult frozen testis sections. We have now clarified this in the Results section as follows (page 12): "A single ODF2-positive mother centriole was detected in 100% (50/50 centriole pairs) of control round spermatids and in 98% (57/58 centriole pairs) of cKO round spermatids".

7. Fig 5A: DAPI is missing in the bottom cKO merged panel
We apologize for the confusion. These images were acquired in the seminiferous tubule lumen, where the nuclei were absent. To clarify this point, we have added the following sentence to the figure legend: "the bottom cKO images were acquired in the seminiferous tubule lumen, where nuclei were absent".

8. ODF2/outer dense fibres are not only in the midpiece, but also in the principal piece. Please update the sentence to reflect this.
This has been corrected.

9. The live cell imaging is very impressive given the constrains of working with this cell type. I think the hypothesis that centrioles move through ICBs is highly plausible but that it has not been directly shown here, I suggest updating 7B to clearly state this is a proposed mechanism/hypothesis.
We thank the Referee for recognizing the technical challenges associated with live-cell imaging of germ cells. We agree that centriole movement through intercellular bridges remains a proposed mechanism that has not been directly demonstrated in this study. Accordingly, we have revised the legend to Fig. 7B to clearly state that this represents a hypothetical model and have also clarified this point in the Discussion section (page 20).

1st Revision - Editorial Decision 19th May 2026

Dear Dr. Takemaru

Thank you for the submission of your revised manuscript to EMBO reports. We have now received the full set of referee reports that is copied below.

As you will see, all referees are very positive about the study and request only minor changes to clarify text and figures.

From the editorial side, there are also a few things that we need before we can proceed with the official acceptance of your study:

- As a standard procedure, we edit the title and abstract of manuscripts to make them more accessible to a general readership and to match them with character and word limitations. Please find the suggested versions below my signature.

- Since you have uploaded image data to the EBI depositroy, please remove the statement 'This study includes no data deposited in external depositories.' from the Data Availability Statement.

- Please rename the "Ethics declarations" to Disclosure and Competing Interests Statement.

- Please doublecheck the author affiliations. For example, affiliation #1 may be incomplete.

- Please remove the legend for EV Table 1 from the manuscript. Also, consider removing Table EV1 in light of the Reagents and Tools table.

- Please remove the instructions from the Reagents and Tools Table.

- Movie EV1 and Movie EV2 have two movie files each, this needs to be corrected. Each movie (one file) needs to be zipped up with its corresponding legend (provided in a text file) so that we have 4 zip folders titled Movie EV1 - Movie EV4. The legends then need to be removed from the manuscript file.

- Please note that EMBO press papers are accompanied online by:
A) a short (2 sentences) summary of the findings and their significance,
B) 2-5 short bullet points highlighting the key results, and
C) a synopsis image in .jpg or .png format that is exactly 550 pixels wide and 300 pixels high. Please note that the text needs to be legible at the final size. Please upload this information along with your revised manuscript (the text for A and B should be provided in one separate Word file uploaded as Cover Art/Synopsis Image).

- Please provide one zip source data folder per figure with one file per panel. Please provide the access for the large deposited source data.

- Please ensure that all figures with n < 5 have individual data points represented. In particular, please doublecheck Figure 3D.

- Our production/data editors have asked you to clarify several points in the figure legends (see below). Please incorporate these changes in the manuscript and return the revised file with tracked changes with your final manuscript submission.
- Please note that the legend for figure 6 is not provided in the sequential manner. This needs to be rectified.
- Please note that the exact p values are not provided in the legend of figure 6G

Kurt Weir
Editor
EMBO Reports

Basal body docking failure triggers centriole clustering and elimination in mammalian spermatogenesis

Cilia are microtubule-based organelles essential for signaling and motility, and their dysfunction causes ciliopathies often associated with infertility. In male germ cells, two types of cilia are present: zygotene primary cilia and sperm flagella. To define the role of cilia in spermatogenesis, we conditionally ablate the distal appendage protein CEP164, required for basal body docking and ciliogenesis, in male germ cells. CEP164 localizes to the mother centriole/basal body throughout spermatogenesis, and its loss leads to male infertility accompanied by absence of both zygotene cilia and sperm flagella. Despite defective ciliogenesis, meiotic chromosome pairing and DNA double-strand break repair proceed normally. However, round spermatids exhibit basal body docking and flagellogenesis defects, and frequently form supernumerary centriole clusters, which appear to be subsequently eliminated via residual bodies. Live-cell imaging reveals that centrioles are highly mobile, and centriole pairs from neighboring cells are often associated possibly through intercellular bridges, forming aggregates. These results establish that basal body docking is crucial for retaining centrioles within spermatids, and its disruption leads to centriole clustering and

loss.

Referee #1:

The authors have responded thoroughly and constructively to my previous comments, and the manuscript is now much stronger as a result. All nine of my original points have been addressed. The title now better reflects the main mechanistic finding (a defect in basal body docking rather than a general loss of cilia). Figure 1D has been replaced with higher-resolution images that more clearly show the abnormal round cells in the cKO caput epididymis. Stage matching across figures has also been improved, with explicit labeling throughout. In addition, the new Figure 5A, which tracks centriole-cluster fate across spermatogenic stages, is a valuable addition that clearly emerged from the revision process. The Methods now include details of the ROSA-EGFP-Centrin1 cross, the contrast artifact in Figure 4D has been corrected, and the mother:daughter centriole ratio has been properly quantified (50/50 in control, 57/58 in cKO) and clearly stated. The absence of DAPI in Figure 5A is now explained by luminal acquisition, ODF2 localization has been corrected to include both midpiece and principal piece, and Figure 7B is now appropriately presented as a hypothetical model.

Taking into account the revisions made in response to the other reviewers, the manuscript has improved considerably overall. These include expanded sampling for meiotic chromosome pairing and DSB repair (Reviewer 1), additional quantification of the manchette and spermatid head (Reviewers 1 and 2), inclusion of a pHH3 mitotic-index control to address potential cell-cycle effects from early Stra8-iCre activity (Reviewer 1), higher temporal resolution live-cell imaging (Reviewer 3), and an expanded Discussion of cilia-independent functions of docked centrioles (Reviewer 3). Together, these additions significantly strengthen the study.

The main remaining limitation-direct visualization of centriole transit through intercellular bridges-is clearly acknowledged in the Discussion, and the corresponding figure and text are appropriately framed. Given the technical challenges described (including poor spermatid viability during live imaging and the limited availability of cKO mice, possibly due to embryonic Stra8-iCre activity), I do not think this experiment can reasonably be required prior to publication. The alternative explanation of de novo centriole amplification is also reasonably ruled out based on the 24-hour live imaging data.

Overall, the manuscript presents three central findings: that zygotene cilia are not required for mouse meiotic chromosome pairing and DSB repair (in contrast to zebrafish), that CEP164 is essential for spermatid basal body docking and flagellogenesis in mammals (unlike in Drosophila), and that undocked centrioles cluster and are subsequently eliminated, potentially via a syncytial mechanism. These conclusions are well supported by the data. The species-specific differences are particularly interesting and should be of broad relevance to the fields of cell and developmental biology, as well as reproductive biology.

A few minor points remain that could be addressed at the proof stage:

-Page 15: "may instead may be eliminated" contains a duplicated "may."
-The additional TEM images included in the response letter (showing a cKO spermatid with four centrioles) would be more useful if incorporated into a supplementary figure rather than remaining only in the rebuttal.
-Figure EV1E combines n=1 animals from three postnatal ages (P20, P22, P23); a brief note in the legend explaining why these time points can be considered equivalent would help readers.

I support publication in EMBO Reports.

Referee #2:

Overview:

Although aspects of spermatogenesis are conserved across animal species, some of the detailed mechanisms are species-specific, even when they involve conserved proteins. An example of this is the ciliary distal appendage protein Cep164, which is dispensable for sperm development in Drosophila and has been reported to be involved in meiotic chromosome pairing and double-strand DNA break repair in zebrafish (although this is controversial). Here, the authors examined the role of Cep164 in mice. Although mouse male germ cells lacking Cep164 undergo meiosis and double-stranded DNA break repair properly, they exhibit later defects in basal body docking and flagellogenesis that is accompanied by formation of supernumerary, highly mobile centrioles that cluster together and are eliminated along with aberrantly structured sperm.

Overall, the revised manuscript is beautifully written. I feel the authors have addressed all the comments of the reviewers. The revisions include additional experiments that were requested for clarity, and the data are clear and compelling.

I have only a short list of minor comments and corrections that can easily be incorporated prior to publication (below).

Text and Figures:

p. 2 (5 lines from bottom of abstract): suggest adding comma after "associated" for clarity
p. 6 (6 lines from bottom of first full paragraph): suggest adding commas surrounding "likely through intercellular bridges: for clarity
p. 7 (line 3 of first full paragraph): the "premature truncation at amino acid 65" is not indicated in Fig. EV1B (it is hinted at in EV1A, but not explicitly shown); adding a vertical arrow or arrowhead with a label to indicate this would be helpful to the reader
p. 8 (6 lines from bottom of first full paragraph): suggest adding "henceforth" before "A-tub" to indicate that this abbreviation will be used throughout the manuscript
p. 9 (6 lines from bottom of upper paragraph): suggest adding "stained" after "adult testis sections"
p. 9 (last two lines): awkward as written; suggest moving "mouse" (currently between "cKO" and "model") to the next line, before "spermatogenesis"
p. 12 (third line from bottom): suggest adding "henceforth" before "G-tub" to indicate that this abbreviation will be used throughout the manuscript
p. 13 (second line of bottom paragraph): suggest replacing "were" with "was"
p. 21 (line 4 of first full paragraph): fix typo in "described"
p. 25 (second line of second full paragraph): add space after "described"
p. 35 (lines 3-6, Figure 4 legend): "Enlarged views of boxed regions are shown below." appears to apply only to the images of cKO round spermatids rather than to the control; suggest moving this sentence after "(yellow arrowheads)."
p. 35 (3 lines from bottom): yellow arrowheads are nearly impossible to see on gray background, especially when printed; suggest changing to red or other contrasting color
p. 38 (second line from bottom of Fig. EV1 legend): suggest changing "mice" to "mouse"

Referee #3:

The revisions made to the manuscript by Chen and colleagues largely address my concerns and have improved the paper. The videos with the 15 sec time intervals nicely capture the difference in stability/movement between the centrioles in round spermatids of control and CEP164 -/- animals. I have a few minor points below. I highly recommend publication after these are addressed. The study does a good job identifying the unexpected consequence of disrupting distal appendages and will be of general interest.
Minor points:
•Both I and reviewer 4 requested descriptions of the ROSA-EGFP-Centrin1 x Cep164fl/fl cross. I do not see this addition in the Methods section. I assume it was an oversight.
•In the description of the original videos (EV1 and 2) in the results section The current draft retains language saying the control "exhibited minimal movement" and the cKO "were highly mobile and dynamic". As discussed previously these conclusions are not supported by this data but are addressed by the new videos. I recommend that the authors instead use EV1 and EV2 to point out that the distance between centriole pairs remains large in the control while the cKO centrioles cluster but do not amplify.
•The second paragraph of the introduction opens with a description of intracellular ciliogenesis which involves vesicle recruitment. It is relevant to this work that cilia can also form through centriole docking to the plasma membrane directly. An appropriate citation for this is Sorokin 1968 (pmid 5661997).

Referee #4:

I am satisfied with the changes that have been made to adequately address my comments. I believe the manuscript is now suitable for publication.

The authors have addressed all minor editorial requests.

Ken-Ichi Takemaru
Stony Brook University
Pharmacological Sciences
United States

Dear Dr. Takemaru,

I am pleased to inform you that your manuscript has been accepted for publication in EMBO reports. Your manuscript will be processed for publication by EMBO Press. It will be copy edited and you will receive page proofs prior to publication. Please note that you will be contacted by Springer Nature Author Services to complete licensing and payment information.

You may qualify for financial assistance for your publication charges - either via a Springer Nature fully open access agreement or an EMBO initiative. Check your eligibility: https://link.springer.com/journal/44319/how-to-publish-with-us

Should you be planning a Press Release on your article, please notify the production team via this form in order to coordinate publication and release dates: https://forms.cloud.microsoft/e/eH4A8JVP6g

If you have any questions, please do not hesitate to contact the Editorial Office. Thank you for your contribution to EMBO Reports.

Yours sincerely,

Kurt Weir
Editor
EMBO Reports

>>> Please note that it is EMBO Reports policy for the transcript of the editorial process (containing referee reports and your response letter) to be published as an online supplement to each paper. If you do NOT want this, you will need to inform the Editorial Office via email immediately. More information is available here: https://link.springer.com/partners/embo-press/editorial-policies#Peer%20review

EMBO Press Author Checklist

Corresponding Author Name: Ken-Ichi Takemaru	
Journal Submitted to: EMBO Reports	
Manuscript Number: EMBOR-2025-63045-T	

USEFUL LINKS FOR COMPLETING THIS FORM
The EMBO Journal - Author Guidelines
EMBO Reports - Author Guidelines
Molecular Systems Biology - Author Guidelines
EMBO Molecular Medicine - Author Guidelines

Reporting Checklist for Life Science Articles (updated January
This checklist is adapted from Materials Design Analysis Reporting (MDAR) Checklist for Authors. MDAR establishes a minimum set of requirements in transparent reporting in the life sciences (see Statement of Task: 10.31222/osf.io/9sm4x). Please follow the journal's guidelines in preparing your
Please note that a copy of this checklist will be published alongside your article.

Abridged guidelines for figures
1. Data
The data shown in figures should satisfy the following conditions:
- ➞ the data were obtained and processed according to the field's best practice and are presented to reflect the results of the experiments in an accurate and unbiased manner.
- ➞ ideally, figure panels should include only measurements that are directly comparable to each other and obtained with the same assay.
- ➞ plots include clearly labeled error bars for independent experiments and sample sizes. Unless justified, error bars should not be shown for technical
- ➞ if n<5, the individual data points from each experiment should be plotted. Any statistical test employed should be justified.
- ➞ Source Data should be included to report the data underlying figures according to the guidelines set out in the authorship guidelines on Data

2. Captions
Each figure caption should contain the following information, for each panel where they are relevant:
- ➞ a specification of the experimental system investigated (eg cell line, species name).
- ➞ the assay(s) and method(s) used to carry out the reported observations and measurements.
- ➞ an explicit mention of the biological and chemical entity(ies) that are being measured.
- ➞ an explicit mention of the biological and chemical entity(ies) that are altered/varied/perturbed in a controlled manner.
- ➞ the exact sample size (n) for each experimental group/condition, given as a number, not a range;
- ➞ a description of the sample collection allowing the reader to understand whether the samples represent technical or biological replicates (including how many animals, litters, cultures, etc.).
- ➞ a statement of how many times the experiment shown was independently replicated in the laboratory.
- ➞ definitions of statistical methods and measures:
  - common tests, such as t-test (please specify whether paired vs. unpaired), simple χ2 tests, Wilcoxon and Mann-Whitney tests, can be unambiguously identified by name only, but more complex techniques should be described in the methods section;
  - are tests one-sided or two-sided?
  - are there adjustments for multiple comparisons?
  - exact statistical test results, e.g., P values = x but not P values < x;
  - definition of 'center values' as median or average;
  - definition of error bars as s.d. or s.e.m.

Please complete ALL of the questions below.
Select "Not Applicable" only when the requested information is not relevant for your study.

Materials

Newly Created Materials	Information included in the manuscript?	In which section is the information available? (Reagents and Tools Table, Materials and Methods, Figures, Data Availability Section)
New materials and reagents need to be available; do any restrictions apply?	Not Applicable	

Antibodies	Information included in the manuscript?	In which section is the information available? (Reagents and Tools Table, Materials and Methods, Figures, Data Availability Section)
For **antibodies** provide the following information:<br>- Commercial antibodies: RRID (if possible) or supplier name, catalogue number and or/clone number<br>- Non-commercial: RRID or citation	Yes	Table EV1

DNA and RNA sequences	Information included in the manuscript?	In which section is the information available? (Reagents and Tools Table, Materials and Methods, Figures, Data Availability Section)
**Short novel DNA or RNA including primers, probes**: provide the sequences.	Not Applicable	

Cell materials	Information included in the manuscript?	In which section is the information available? (Reagents and Tools Table, Materials and Methods, Figures, Data Availability Section)
**Cell lines:** Provide species information, strain. Provide accession number in repository **OR** supplier name, catalog number, clone number, and/**OR** RRID.	Not Applicable	
**Primary cultures:** Provide species, strain, sex of origin, genetic modification status.	Not Applicable	
Report if the cell lines were recently **authenticated** (e.g., by STR profiling) and tested for mycoplasma contamination.	Not Applicable	

Experimental animals	Information included in the manuscript?	In which section is the information available? (Reagents and Tools Table, Materials and Methods, Figures, Data Availability Section)
**Laboratory animals or Model organisms:** Provide species, strain, sex, age, genetic modification status. Provide accession number in repository **OR** supplier name, catalog number, clone number, **OR** RRID.	Yes	Materials and Methods
**Animal observed in or captured from the field:** Provide species, sex, and age where possible.	Not Applicable	
Please detail **housing and husbandry conditions**.	Yes	Materials and Methods

Plants and microbes	Information included in the manuscript?	In which section is the information available? (Reagents and Tools Table, Materials and Methods, Figures, Data Availability Section)
**Plants:** provide species and strain, ecotype and cultivar where relevant, unique accession number if available, and source (including location for collected wild specimens).	Not Applicable	
**Microbes:** provide species and strain, unique accession number if available, and source.	Not Applicable	

Human research participants	Information included in the manuscript?	In which section is the information available? (Reagents and Tools Table, Materials and Methods, Figures, Data Availability Section)
If collected and within the bounds of privacy constraints report on age, sex and gender or ethnicity for all study participants.	Not Applicable	

Core facilities	Information included in the manuscript?	In which section is the information available? (Reagents and Tools Table, Materials and Methods, Figures, Data Availability Section)
If your work benefited from core facilities, was their service mentioned in the acknowledgments section?	Yes	Materials and Methods, Acknowledgements

Design

Study protocol	Information included in the manuscript?	In which section is the information available? (Reagents and Tools Table, Materials and Methods, Figures, Data Availability Section)
If study protocol has been **pre-registered, provide DOI in the manuscript**. For clinical trials, provide the trial registration number **OR** cite DOI.	Not Applicable	
Report the **clinical trial registration number** (at ClinicalTrials.gov or equivalent), where applicable.	Not Applicable	

Laboratory protocol	Information included in the manuscript?	In which section is the information available? (Reagents and Tools Table, Materials and Methods, Figures, Data Availability Section)
Provide DOI OR other citation details if **external detailed step-by-step protocols** are available.	Yes	Materials and Methods

Experimental study design and statistics	Information included in the manuscript?	In which section is the information available? (Reagents and Tools Table, Materials and Methods, Figures, Data Availability Section)
Include a statement about **sample size** estimate even if no statistical methods were used.	Yes	Materials and Methods, Figure legends
Were any steps taken to minimize the effects of subjective bias when allocating animals/samples to treatment (e.g. **randomization procedure**)? If yes, have they been described?	Not Applicable	
Include a statement about **blinding** even if no blinding was done.	Yes	Materials and Methods
Describe **inclusion/exclusion criteria** if samples or animals were excluded from the analysis. Were the criteria pre-established?<br><br>If sample or data points were omitted from analysis, report if this was due to attrition or intentional exclusion and provide justification.	Not Applicable	
For every figure, are **statistical tests** justified as appropriate? Do the data meet the assumptions of the tests (e.g., normal distribution)? Describe any methods used to assess it. Is there an estimate of variation within each group of data? Is the variance similar between the groups that are being statistically compared?	Yes	Materials and Methods, Figure legends

Sample definition and in-laboratory replication	Information included in the manuscript?	In which section is the information available? (Reagents and Tools Table, Materials and Methods, Figures, Data Availability Section)
In the figure legends: state number of times the experiment was **replicated** in laboratory.	Yes	Figure legends
In the figure legends: define whether data describe **technical or biological replicates**.	Yes	Figure legends

Ethics

Ethics	Information included in the manuscript?	In which section is the information available? (Reagents and Tools Table, Materials and Methods, Figures, Data Availability Section)
Studies involving **human participants**: State details of **authority granting ethics approval** (IRB or equivalent committee(s), provide reference number for approval.	Not Applicable	
Studies involving **human participants**: Include a statement confirming that **informed consent** was obtained from all subjects and that the experiments conformed to the principles set out in the WMA Declaration of Helsinki and the Department of Health and Human Services Belmont Report.	Not Applicable	
Studies involving **human participants:** For publication of **patient photos**, include a statement confirming that consent to publish was obtained.	Not Applicable	
Studies involving experimental **animals**: State details of **authority granting ethics approval** (IRB or equivalent committee(s), provide reference number for approval. Include a statement of compliance with ethical regulations.	Yes	Materials and Methods
Studies involving **specimen and field samples:** State if relevant **permits** obtained, provide details of authority approving study; if none were required, explain why.	Not Applicable	

Dual Use Research of Concern (DURC)	Information included in the manuscript?	In which section is the information available? (Reagents and Tools Table, Materials and Methods, Figures, Data Availability Section)
Could your study fall under dual use research restrictions? Please check biosecurity documents and list of **select agents and toxins** (CDC): https://www.selectagents.gov/sat/list.htm	Not Applicable	
If you used a select agent, is the security level of the lab appropriate and reported in the manuscript?	Not Applicable	
If a study is subject to dual use research of concern regulations, is the name of the **authority granting approval and reference number** for the regulatory approval provided in the manuscript?	Not Applicable	

Reporting

The MDAR framework recommends adoption of discipline-specific guidelines, established and endorsed through community initiatives. Journals have their own policy about requiring specific guidelines and recommendations to complement MDAR.

Adherence to community standards	Information included in the manuscript?	In which section is the information available? (Reagents and Tools Table, Materials and Methods, Figures, Data Availability Section)
State if relevant guidelines or checklists (e.g., **ICMJE, MIBBI, ARRIVE, PRISMA**) have been followed or provided.	Yes	Materials and Methods
For **tumor marker prognostic studies,** we recommend that you follow the **REMARK** reporting guidelines (see link list at top right). See author guidelines, under 'Reporting Guidelines'. Please confirm you have followed these guidelines.	Not Applicable	
For **phase II and III randomized controlled trials**, please refer to the **CONSORT** flow diagram (see link list at top right) and submit the CONSORT checklist (see link list at top right) with your submission. See author guidelines, under 'Reporting Guidelines'. Please confirm you have submitted this list.	Not Applicable	

Data Availability

Data availability	Information included in the manuscript?	In which section is the information available? (Reagents and Tools Table, Materials and Methods, Figures, Data Availability Section)
Have **primary datasets** been deposited according to the journal's guidelines (see 'Data Deposition' section) and the respective accession numbers provided in the Data Availability Section?	Yes	Data Availability Section
Were **human clinical and genomic datasets** deposited in a public access-controlled repository in accordance to ethical obligations to the patients and to the applicable consent agreement?	Not Applicable	
Are **computational models** that are central and integral to a study available without restrictions in a machine-readable form? Were the relevant accession numbers or links provided?	Not Applicable	
If publicly available data were reused, provide the respective **data citations in the reference list**.	Not Applicable	
